# Vectorial capacities for malaria in eastern Amazonian Brazil depend on village, vector species, season, and parasite species

**DOI:** 10.1186/s12936-022-04255-x

**Published:** 2022-08-16

**Authors:** Robert H. Zimmerman, Allan K. R. Galardo, L Philip Lounibos, Clicia Galardo, A. Kadir Bahar, Edzard van Santen

**Affiliations:** 1 Florida Medical Entomology Laboratory, University of Florida/IFAS, 200 9th Street SE, Vero Beach, FL 32962 USA; 2 Laboratório de Entomologia Médica, Instituto de Pesquisas Científicas E Tecnológicas Do Estado de Amapá-IEPA, Campus do IEPA da Fazendinha-CEP, Macapá, 68912-250 Brazil; 3 Department of Educational Psychology, Athens, GA USA; 4 Agronomy Department and Statistical Consulting Unit, University of Florida/IFAS, Gainesville, FL 32611 USA

**Keywords:** *Anopheles*, Vectorial capacity, Malaria, Amazon

## Abstract

**Background:**

The vector species in the Amazon River Basin are regionally and locally diverse, which makes it imperative to understand and compare their roles in malaria transmission to help select appropriate methods of intervention and evaluation. The major aim of this study was to measure the vectorial capacity of five *Anopheles* species in three neighbouring villages, for two *Plasmodium* parasite species affecting humans.

**Methods:**

From 32 consecutive months of sampling in three villages, 1.5–7.0 km apart, on the Matapi River, Amapá State, Brazil, vectorial capacities (C) were estimated as time series for *An*. *darlingi*, *An*. *marajoara*, *An*. *nuneztovari*, *An*. *triannulatus*, and *An*. *intermedius*. Monthly parity measurements for each vector species were used to estimate daily survivorship and compared to estimates of survivorship from mark-release-recapture experiments. Gonotrophic cycle lengths were estimated through a time-series analysis of parity data, and durations of sporogony at study site temperatures for the two malaria parasite species were estimated from previous literature.

**Results:**

The absolute abundances of five vector species were strongly tracked by the spatial variation in C among villages. Temporally, C varied between wet and dry seasons, with *An. darlingi*, *An. marajoara* and *An. triannulatus* exhibiting higher C in the dry season from August to December, and *An. nuneztovari* its highest C early in the rainy season in January and February. *Anopheles intermedius* exhibited higher C in the rainy season from April to June than in the dry season. Significant differences in overall survival for each independent variable, and a significant difference in C between wet and dry seasons, among villages, and among vector species for both *Plasmodium falciparum* and *Plasmodium vivax.* A generalized linear mixed model (GLMM) analysis by village showed significant effects of vector species on C in only one village, but significant effects of parasite species in all three. Although the GLMM analysis detected no significant parasite x vector species interaction effects on C, effects on C of spline regressions of C dynamics x vector species interactions were significant in all villages.

**Conclusions:**

These detailed analyses of entomological and parasitological variables revealed hidden complexities of malaria epidemiology at local scales in neighbouring riverine villages of the Amazon Region.

**Supplementary Information:**

The online version contains supplementary material available at 10.1186/s12936-022-04255-x.

## Background

The Amazon River Basin has the highest incidence of malaria in the Americas and the highest population at risk, and this disease has recently increased in several countries [1]. Brazil has had recent success in lowering malaria incidence in several states in the Amazon region, but it is still a serious public health problem [2]. Present-day and new interventions need to be evaluated at the local level and on an eco-regional scale where vector heterogeneity and distribution are essential components along with environmental determinants, landscape patterns, and human distribution [3, 4]. The evaluation and integration of new intervention strategies and existing methods, such as impregnated bed nets, vaccines, and transmission-blocking immunity in mosquitoes depend on accurate indicators of change in malaria incidence. Many times, clinical diagnosis is not sufficient to observe changes in malaria transmission, particularly when (a) malaria transmission is low, (b) *Plasmodium vivax* is the predominant parasite, and (c) a high percentage of asymptomatic malaria cases exists, as is the case in South America [5–10].

The regional and local heterogeneity of vector species in the Americas adds another dimension to the understanding of malaria transmission dynamics [3, 11–15]. The rate at which a mosquito vector becomes infected and transmits malaria to people depends on several parameters related to the vector’s life history. They include the ratio of mosquitoes to human density, the human-biting habits, duration of the gonotrophic cycle, mosquito survival, and the length of the sporogonic cycle of the *Plasmodium* parasite in the vector. These parameters are components of the vectorial capacity (C) equation [16], which is defined as “a daily rate of potentially infective contact between persons through the vectors (here “potentially infective” refers to the survival of the vector through the incubation period or “extrinsic cycle”)” [17].

Previous research on a vector’s capacity to transmit malaria was focused on the analysis of the assumptions, components, and need for the vectorial capacity approach in malaria prevention and control. Researchers have questioned whether all these components need to be measured, or only the dominant entomological variables are necessary for epidemiological predictions [18]. Burkot and Graves [19] suggested that although vectorial capacity serves as a relative estimate of transmission, more accurate estimates may reduce bias in the measurement of the entomological parameters. The entomological inoculation rate (EIR, the product of human biting rate and the proportion of infected mosquitoes), and malaria prevalence in children were shown to be strongly correlated in Papua New Guinea [20]. However, in the present study area differences in EIRs were more dependent on anopheline abundance than on sporozoite rates [8].

A cyclical model for the determination of vectorial capacity from vector infection rates, which does not depend on constant feeding frequency or age-independent survival was examined [21]. In principle, it would give better estimates of transmission where there is heterogeneity in the vector population, in the environment, or in age dependent survival. However, the cyclical model can be used only if the vector population is large, engorged mosquitoes are easily found, and the population has a high infection rate [21]. These conditions are uncommon in the Americas [10].

In the Brazilian Amazon, the entomological correlates associated with malaria incidence have been collected [14, 22–25]. Also, recent interest has been in reexamining the components of vector competence, reproductive rate, and vectorial capacity to broaden the understanding of the theory of vector dynamics [26]. This approach was put into practice for the Neotropical vector, *Anopheles darlingi* (= *Nyssorhynchus darlingi*) [27].

In the present study, survival rates and gonotrophic cycle lengths of five vector species were measured in a malaria endemic area where multiple vectors were involved in malaria transmission. They were combined with the results of previous research carried out in the same area [8, 14, 24, 28]. The major aim of this study was to measure C and its natural variation among species, among villages, and between seasons and parasite species in relation to malaria transmission.

## Methods

### Study sites

**Fig. 1:**
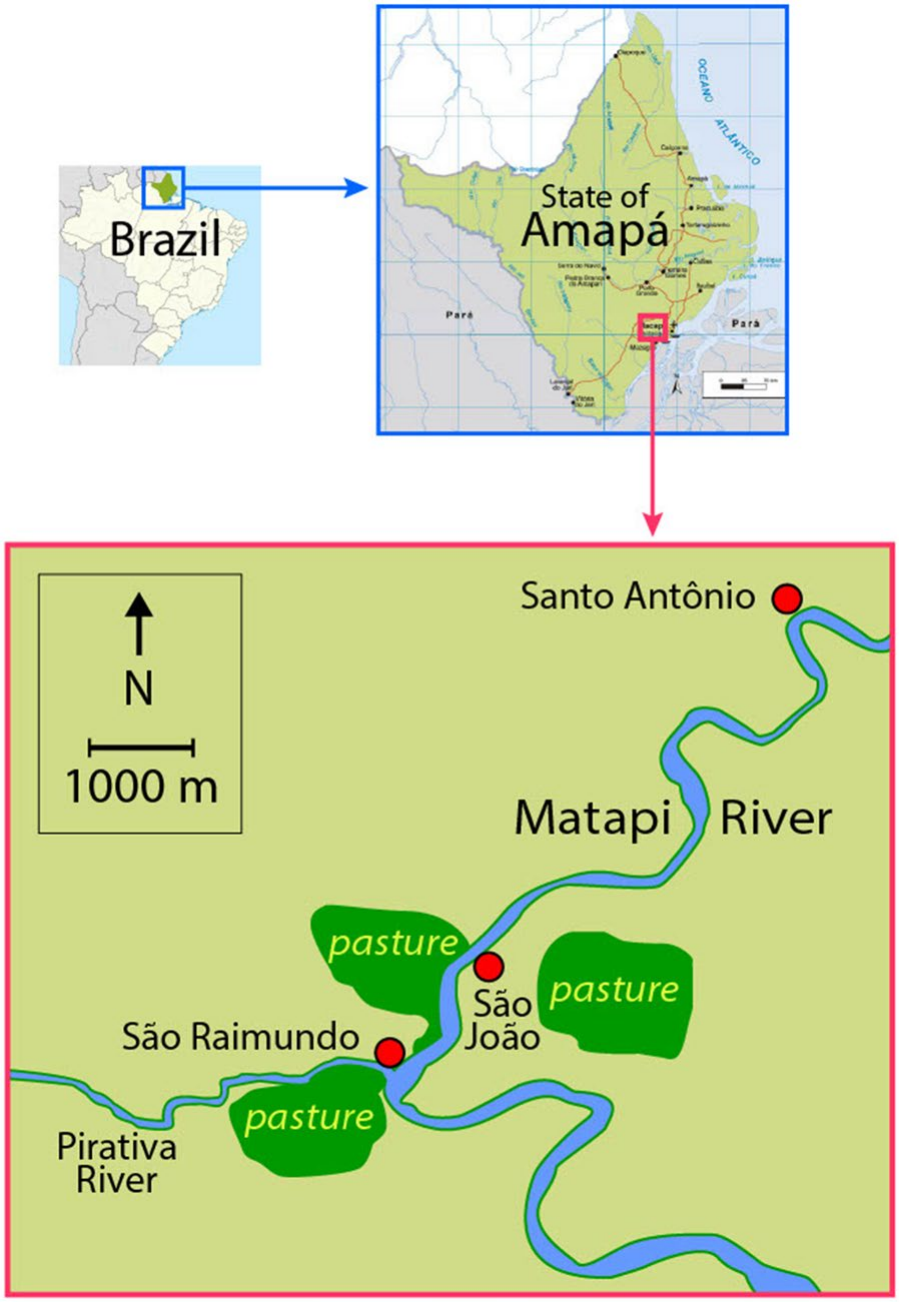
Map showing the three riverine communities where collections were made

Three communities separated by 1.5 to 7.0 km, along the Matapí River, Amapá State, Brazil, were selected for study; São Raimundo (00°02′ N; 051°15′ W), São João (00°02′ N; 051°14′ W) and Santo Antônio (00°05′ N; 051°12′W) (Fig. 1). A description of the region was presented by Zimmerman et al. [28]. The climate is hot and humid (mean relative humidity 85%) with temperatures ranging from 22 to 32 °C. The wet season extends from January to July (mean seasonal precipitation 2100 mm), and the dry season from August to December (mean seasonal precipitation 178 mm). The ecosystem is a mixed flooded forest (*várzea*)—marsh habitat with many small streams (*igarapés*) draining into the Pirativa and the Matapí River systems. The freshwater level rises and falls with the daily tides, flooding the forest floor during high tide, particularly during the wet season. Malaria is endemic in the area, with *Plasmodium falciparum*, *P. vivax* VK210, *P. vivax* VK247, and *Plasmodium malariae* detected in five *Anopheles* species [8]. *Plasmodium malariae* was not included in this study because no monoclonal antibodies were available for testing this parasite species after March 2004.

### Mosquito collection

A complete description of mosquito collection procedures is given in Galardo et al. [8]. Briefly, mosquitoes were aspirated as they landed on one exposed leg of a collector and placed in screened 0.5L ice cream cartons modified as cages. Mosquitoes in cages were placed in a Styrofoam® cooler with wet paper towels and transported to the field laboratory every hour until 22:30 h; after 23:30 h they were brought to the laboratory in the morning. They were anesthetized with ethyl acetate, and identified to species using the keys of Consoli and Lourenço de Oliveira [29]. Collections were made over 32 consecutive months from April 2003 until November 2005.

### Survival studies

The daily survival rate *(p*) of a vector is a key parameter in the vectorial capacity model *ma*^*2*^*p*^*n*^*/-ln(p*) (see explication of equation in the Vectorial Capacity section, below) [16]. It is the fraction of the vector population that survives the extrinsic incubation period (EIP) as *p*^*n*^ where *n* = the duration of the sporogonic cycle in days and is included in the formula for the expectation of life (1/*-ln(p*)) in the denominator. The assumption is that *p* is independent of age. Thus, vector biologists and malaria researchers have employed various methods to measure the daily survival rate of anophelines, including mark-release-recapture (MMR), changes in follicular development, time series analysis, and parous rates to quantify vector survival and to better understand their role in malaria transmission [23, 30–35]; each method has its limitations. Different methods were used to determine which method would be best to calculate and compare C values of the vectors present in the study area and in future studies.

Three different methods were examined: MRR, Davidson’s parity, and time-series analysis. For the Davidson method and the time-series analysis, collected females were identified to species and dissected immediately after capture or if necessary, held until the next morning in a refrigerator (4 °C) to retard ovarian development. Paired ovaries were dissected out of female mosquitoes and identified as parous or nulliparous using the method of Detinova [36]. Parous rates were measured by dividing the total number of parous mosquitoes collected from human landing catches (HLC) by the total number of parous and nulliparous females collected. Parity was calculated by site, species and date of capture. The mark-release recapture study and the time series analysis of parity data were conducted only in São João.

#### Mark-release-recapture (MRR) studies

MRR studies were conducted to measure the anopheline survival rates using the “trellis” method of Fisher-Ford [34, 37]. Mosquitoes were captured from HLC and placed in liter size containers (25 mosquitoes per container), lightly anesthetized with CO_2_, identified to species, and placed in species-specific liter containers in which they were lightly dusted with Day-Glo® fluorescent powder applied with an insufflator. A subset of 25 dusted and 25 non-dusted mosquitoes were kept in separate containers to observe survivorship. Dusted mosquitoes were released the same night of capture from a central release point in the village. Mosquitoes that did not fly out of the container were subtracted from the release count.

Captured mosquitoes were marked with different coloured dusts for five consecutive nights. After the first marking, all collected mosquitoes were anesthetized and examined under a stereomicroscope with ultraviolet (UV) illumination to see if they were previously marked. Marked mosquitoes were classified according to trophic stage; unfed, engorged, and gravid [36]. Mark-recapture experiments were conducted twice a year in the wet and dry season for two years. Marked mosquitoes were released for five consecutive nights during the first three releases and recaptured for 9 days after the initial release. In the fourth release mosquitoes were marked every other day and recaptured six times over 9 days.

#### Davidson method

Monthly 3-day all-night HLCs in each village were used to capture mosquitoes for calculations of parity and survival rates. Daily survivorship rate *p* was estimated by the formula of Davidson [38] where *p* = *(PR)*^*1/GC*^; *PR* is the parous rate and *GC* is the length of the gonotrophic cycle measured in days. Three and 3.4-day gonotrophic cycle lengths were selected for the analysis of survival rate based on results of previous field studies with *An. darlingi*, and the results of the 32-day time series analysis for *An*. *darlingi*, *An*. *marajoara*, *An*. *intermedius*, and *An. nuneztovari*. In Brazil, the gonotrophic cycle length for *An*. *darlingi* was estimated to be between 2.19 and 3 days or greater [23, 30, 32, 33, 39]. In Venezuela, a 4-day gonotrophic cycle length was used to calculate C for *An. nuneztovari*, *An*. *marajoara* (as *An. albitarsis*), and *An*. *triannulatus* but was based on laboratory estimations [40]. The Davidson method also was used to compare survival rates during the MRR study and the time series analysis.

#### Time-series analysis

A time series analysis of parous rate data was used to estimate the gonotrophic cycle length and survival rates of the five species studied. Data from the 32-day captures in the wet and dry seasons from the village of São João (n = 4) were examined using a time-series method described by Birley and Boorman [31], applied to anophelines [41, 42], and improved by Holmes and Birley [43]. The survival rate analysis method of Holmes and Birley [43] that incorporated weighted linear regression and filtered cross-correlation was applied. The following equations were used:

$$P_{t} = S \cdot T_{t - u}$$

$$T_{t} = P_{{\text{t}}} + {\text{ N}}_{{\text{t}}}$$

where N_t_ and *P*_t_ are the number of nulliparous and parous mosquitoes on day *t, u* is the mean gonotrophic cycle length, *T*_*t-u*_ is the total number of nulliparous and parous mosquitoes collected on day *t-u*, and *S* is equal to the proportion surviving one gonotrophic cycle length. The equation *P*_t_ = *S·T*_*t-u*_ is then treated as a linear regression of *P*_*t*_ on *T*_*t-u*_. The regression slope is an estimate of S. A cross correlation index (CCI) analysis of the time series between the number of nulliparous (N_t_), parous (P_t_) and total mosquitoes (*T*_*t*_) was used to determine significant peaks in CCI. The value *u* is equal to the lag period of these peaks (see [43]). The value *u* is then chosen, which maximizes the cross-correlation. The daily survival rate was calculated using the formula *p* = *P*^*1/u*^ where *p* = daily survival rate, *P* = the survival rate for the mean gonotrophic period and *u* is the estimated gonotrophic cycle length in days.

### Vectorial capacity

The method developed by Garrett-Jones [16] was used to measure and compare vectorial capacity among vector species and villages and between malaria parasites and seasons. The C is calculated by this equation:

$$C = ma^{2} p^{n} /- ln(p)$$

where *ma* is the number of female mosquitoes per person per night; *a* is the human-biting frequency per day, which is estimated as the human biting index divided by the gonotrophic cycle (GC); *p* is the daily survival rate of the vector, which is assumed to be constant and estimated from the formula *p* = ^*GC*^***√*** parity; and *n* = the duration of the sporogonic cycle in days [40]. The duration of the sporogonic cycle was calculated using the formula *n* = *T/(t—t*_*min*_) where *n* = duration of the sporogonic cycle, *T* = 111 for *P. falciparum* and 105 for *P. vivax, t* = average temperature in degrees centigrade, and min = 16 for *P. falciparum* and 14.5 for *P. vivax* [44].

Temperature was recorded daily using HOBO data loggers (Onset Computer Corporation, Bourne, MA, USA) placed in São Raimundo. The human biting rate (*ma*) and the human biting index (*a*) used to calculate C were from previous research conducted in the same study area [14, 28]. Two genotypes of vivax malaria, *P. vivax* 247 and *P. vivax* 210, are known from the Amazon region of Brazil [45] and were distinguished in anophelines collected at the study sites [8]. They were pooled together as *P. vivax* for the analysis of C.

### Statistical analysis

Generalized linear mixed model (GLMM) procedures as implemented in SAS® PROC GLIMMIX (SAS/STAT 15.1, SAS Institute, Cary, NC) were used to analyse the data. Survival rates were modelled using a beta distribution function, which can account for overdispersion in the data, using the fixed effects model Village + Species + Season + Village × Species + Village × Season + Species × Season + Village × Species × Season. Values of zero or two or fewer mosquitoes were excluded from analyses. Post-hoc least square means (LSMs) of survivorship between seasons (wet vs. dry) were compared for each Village × Species interaction by Fisher’s least significant difference (LSD) test. Means plus 95%CI were back-transformed to the data scale for reporting purposes.

Similarly, C values were analysed with the same PROC but using a lognormal distribution function. Data were analysed by village using the fixed effects model Response = Species + Parasite + Species × Parasite + spline (MAI) × Species, where the spline regression was calculated from a cubic truncated power function based on month after the initiation (MAI) of sampling. Knots were chosen by visual inspections of the graphed raw data. The repeated nature of the C estimates through R-side modelling was accounted for using a first-order autoregressive covariance structure. Because the Species × Parasite interaction was never significant (p > 0.71) we calculated least squares means (LSMs) of each species for each MAI and compared species within each village and MAI using a simple pairwise *t*-test at α = 0.01 based on the arguments for pairwise comparisons [46, 47]. The non-significant Species × Parasite interaction justified calculation of a mean C derived from averaging C of the two parasite species calculated separately.

Spearman correlation coefficients (ρ) were used to measure the strength and direction of the relationships between vectorial capacities and mosquito abundances for each vector species during the 32-month collection period.

## Results

### Survival rates

#### Fisher-Ford mark release recapture (MRR)

Four MRR experiments were conducted in São João village; two during the wet season and two during the dry season. Three species, *An. darlingi*, *An. marajoara* and *An. nuneztovari* were captured in sufficient numbers for the experiments (Additional file 1: Tables S1–S4). The number of marked and released mosquitoes ranged from 491 for *An. marajoara* in October 2004 to 6061 for *An. nuneztovari* in May–June 2004. The recapture rates for *An. darlingi* were 1.8% in May–June 2004, 2.5% in October 2004, 2.8% in June 2005, and 0.9% in September 2005. The first recaptures of this species were collected on day 1 after release and the last were collected on day 8 after release. Ten recaptured females had blood from less than 12 h to 48 h old in their abdomens, which represented 11.2% of the total mosquitoes recaptured. No gravid females were captured. The recapture rates for *An. marajoara* were 1.1% in May–June 2004, 1.2% in October 2004, 1.9% in June 2005, and 1.9% in September 2005. The first recaptures of this species were collected on day 1 after release and the last were collected on day 9 after release. Nine recaptured *An. marajoara* had blood from less than 12 h to 48 h old in their abdomen when recaptured, which represented 9.6% of the recaptures of this species. Two females had retained one egg in an ovariole, and one had six eggs. The recapture rates for *An. nuneztovari* were 0.7% in May–June 2004, 0.3% in October 2004, 0.6% in June 2005, and 0.4% in September 2005. The first recaptures of this species were collected on day 1 after release and the last were collected on day 9 after release. Nine recaptured *An. nuneztovari* had blood from less than 12 h to over 48 h old in their abdomen, which represented 13.8% of the recaptures of this species. No females of this species were gravid (Additional file 1: Tables S1–S4).

The daily survival rates varied among species, method of estimation, and between seasons (Table 1). *Anopheles marajoara* had the lowest Fisher-Ford survival rates except for September 2005 when *An. nuneztovari* had a daily survival rate of 0.15, and *An. nuneztovari* had the highest Fisher-Ford rates of 0.94 in June 2004 and 0.98 in June 2005. The daily survival rates of marked mosquitoes held in laboratory cages were 0.88 for *An. darlingi*, 0.83 for *An. marajoara* and 0.87 for *An. nuneztovari*. No differences in survival rates were observed between marked and unmarked mosquitoes held in cages.

**Table 1:** Daily survival rates of *An. darlingi*, *An. marajoara* and *An. nuneztovari* using the Fisher & Ford mark-release-recapture (MRR) trellis method (1947) and Davidson method (1954)

Species	Date	Fisher & Ford	Davidson			
			MRR^a^		3-day^b^	
		MRR	GC = 3	GC = 3.4	GC = 3	GC = 3.4
*An. darlingi*	Jun-04	0.55	0.82	0.84	0.73	0.76
*An. darlingi*	Oct-04	0.71	0.89	0.90	0.86	0.88
*An. darlingi*	Jun-05	0.72	0.71	0.74	0.70	0.73
*An. darlingi*	Sept-05	0.39	0.83	0.85	0.74	0.77
*An. marajoara*	Jun-04	0.43	0.80	0.82	0.75	0.77
*An. marajoara*	Oct-04	0.20	0.78	0.80	0.88	0.89
*An. marajoara*	Jun-05	0.25	0.61	0.65	0.76	0.78
*An. marajoara*	Sept-05	0.31	0.75	0.77	0.71	0.73
*An. nuneztovari*	Jun-04	0.94	0.85	0.87	0.78	0.81
*An. nuneztovari*	Oct-04	0.56	0.85	0.87	0.87	0.88
*An. nuneztovari*	Jun-05	0.98	0.69	0.72	0.66	0.69
*An. nuneztovari*	Sept-05	0.15	0.79	0.81	0.74	0.76

GC, gonotrophic cycle (length in days)

^a^Daily survival rates of mosquitoes collected during the last 5 days of the MRR study

^b^Daily survival rates of mosquitoes captured in the monthly 3-day collection during the MRR study

#### Davidson method

Daily survival rates calculated by the Fisher & Ford trellis method were lower than the survival rates calculated by the Davidson method from unmarked mosquitoes collected during the last five days of the MRR study and the routine 3-day collection for the time period during the MMR study on 9 of 12 dates for GC = 3 and 10 of 12 dates for GC = 3.4 (Table 1). Daily survival rates of mosquitoes using the Davidson method for the MMR and 3-day method were higher in the dry season than in the wet season for *An. darlingi*, equal or higher in the dry season for *An. nuneztovari*, and variable for *An. marajoara*. Collections of *An. triannulatus* by HLC were extremely low in the wet season and low for *An. intermedius* in the dry season during the MRR periods [14], therefore we decided not to calculate daily survival rates of these species during these periods using the Davidson method. However, daily survival rates were calculated using the Davidson method for the monthly 3-day collections over 32 months for all five species (Additional file 2: Tables S1–S5). The mean survival rates between the wet and dry seasons showed that the survival rates of all five species were higher in the dry season than in the wet season, with differences ranging from 0.01 to 0.15, except for *An*. *intermedius* in Santo Antônio where the wet season survival rate was slightly higher (Additional file 3: Table S1).

**Fig. 2:**
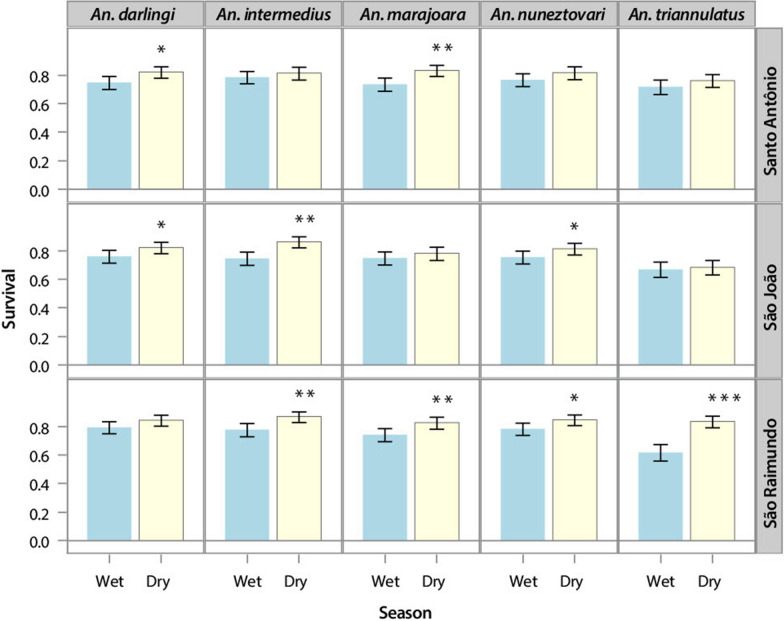
Comparison of survival rates between seasons by village and species. Blue bars = rainy season, yellow bars = dry season. The mean (± 95% CI) differences are significant at p < 0.05 (*), p < 0.01(**) or p < 0.001(***)

The 3-way ANOVA revealed significant variation in survivorship rates, calculated by parous rates, between seasons, among species and villages (Table 2). The season × village interaction showed significant variation in survivorship rates (p = 0.0345), the species x village interaction was not significant (p = 0.675), and the three-way interaction between season x species x village was not significant (p = 0.0715). The consistency of superior dry-season survivorship among species accounts for the non-significant species × season interaction (Table 2, Fig. 2). The significant village × species interaction is reflected in the differences among villages in significant differences between wet and dry LSMs of survivorship (Fig. 2), which shows that São Raimundo was more likely than São João and Santo Antônio to have significant differences between seasons in survivorship for multiple vector species.

**Table 2:** Results of the 3-way ANOVA for the effects of season, Anopheline species, and village on survivorship

Effect	*df *(num)	*df *(den)	F-value	*p*-value
Village	2	423	4.8	0.0086
Species	4	423	17.3	< 0.0001
Species x Village	8	423	0.7	0.6754
Season	1	423	74.9	< 0.0001
Season x Village	2	423	3.4	0.0345
Species x Season	4	423	0.2	0.9396
Species x Season x Village	8	423	1.8	0.0715

#### Time-series analysis

Survivorship was calculated by the time series method of Holmes & Birley [43] for four species (Table 3), excluding *An*. *triannulatus* because too few individuals of this species were collected. Survival rates per gonotrophic period (*P*) and the daily survival rate (*p*) were lowest for *An. marajoara* during all four experiments. The highest *P* and *p* were calculated for *An. nuneztovari* in March 2004. Daily survival rates varied between collection periods. The daily survival rates calculated by the Davidson method using the gonotrophic cycle length from the time series (*u*) were similar to the survival rates calculated from the time series analysis.

**Table 3:** Results of the time series analysis over four 32-day capture periods and the daily survival rates estimated from the formula of Davidson (1954) during the same time period

Species	Date	*P*^a^	SE^b^	u^c^	CCI^d^	p = P^(1/u) e^	Davidson p^f^	3-day p^g^
*An. darlingi*	Mar-04	0.52	0.02	3.42	0.62	0.83	0.82	0.77
*An. darlingi*	Sep-04	0.56	0.01	3.49	0.97	0.85	0.85	0.83
*An. darlingi*	Mar-05	0.36	0.03	3.33	0.95	0.74	0.77	0.69
*An. darlingi*	Oct-05	0.51	0.01	3.37	0.91	0.82	0.85	0.83
*An. marajoara*	Mar-04	0.41	0.01	3.58	0.69	0.78	0.77	0.76
*An. marajoara*	Sep-04	0.39	0.01	3.55	0.96	0.77	0.76	0.80
*An. marajoara*	Mar-05	0.33	0.01	3.47	0.95	0.73	0.73	0.80
*An. marajoara*	Oct-05	0.34	0.02	3.06	0.42	0.73	0.78	0.77
*An. nuneztovari*	Mar-04	0.78	0.01	3.53	0.71	0.93	0.85	0.79
*An. nuneztovari*	Sep-04	0.54	0.01	3.50	0.81	0.84	0.84	0.87
*An. nuneztovari*	Mar-05	0.43	0.01	3.52	0.95	0.79	0.79	0.71
*An. nuneztovari*	Oct-05	0.57	0.02	3.47	0.10	0.85	0.86	0.95
*An. intermedius*	Mar-04	0.52	0.01	3.60	0.67	0.83	0.82	0.59
*An. intermedius*	Sep-04	0.51	0.01	3.44	0.33	0.82	0.83	0.77
*An. intermedius*	Mar-05	0.36	0.02	3.45	0.69	0.74	0.76	0.56

^a^ *P* = survival rate per gonotrophic cycle length, ^b^ SE = standard error, ^c^ u = mean length of the gonotrophic cycle in days, ^d^ CCI = cross correlation index, ^e^ p = *P*^1/u^ = daily survival rate, ^f^ Davidson p = the survival rate using Davidson method over the 32-day period and u, ^g^ 3-day p = 3-day HLC survival rate using Davidson method and u

*Anopheles marajoara* had the lowest survival rates, and *An*. *intermedius* had equal or lower survival rates compared to *An. darlingi* and *An. nuneztovari* (Table 3). Daily survival rates in the dry months (Sept-04, Oct-05) compared to the wet months (March-04, March-05) during the MRR, were variable; only *An. darlingi* had higher survival rates in the dry months than in the wet months for all three estimates. The lengths of the gonotrophic cycles (*u*) of a species varied over the study period (Table 3). The lowest estimate of *u* was for *An. marajoara,* 3.06 days in October 2005, and the highest was for *An. intermedius*, 3.60 days in March 2004 (Table 3). The gonotrophic cycle length for *An. intermedius* was not calculated for October 2005 because only 18 mosquitoes were collected over the 32-day period, and too few *An. triannulatus* were collected during the study to calculate its gonotrophic cycle length.

### Vectorial capacity

**Fig. 3:**
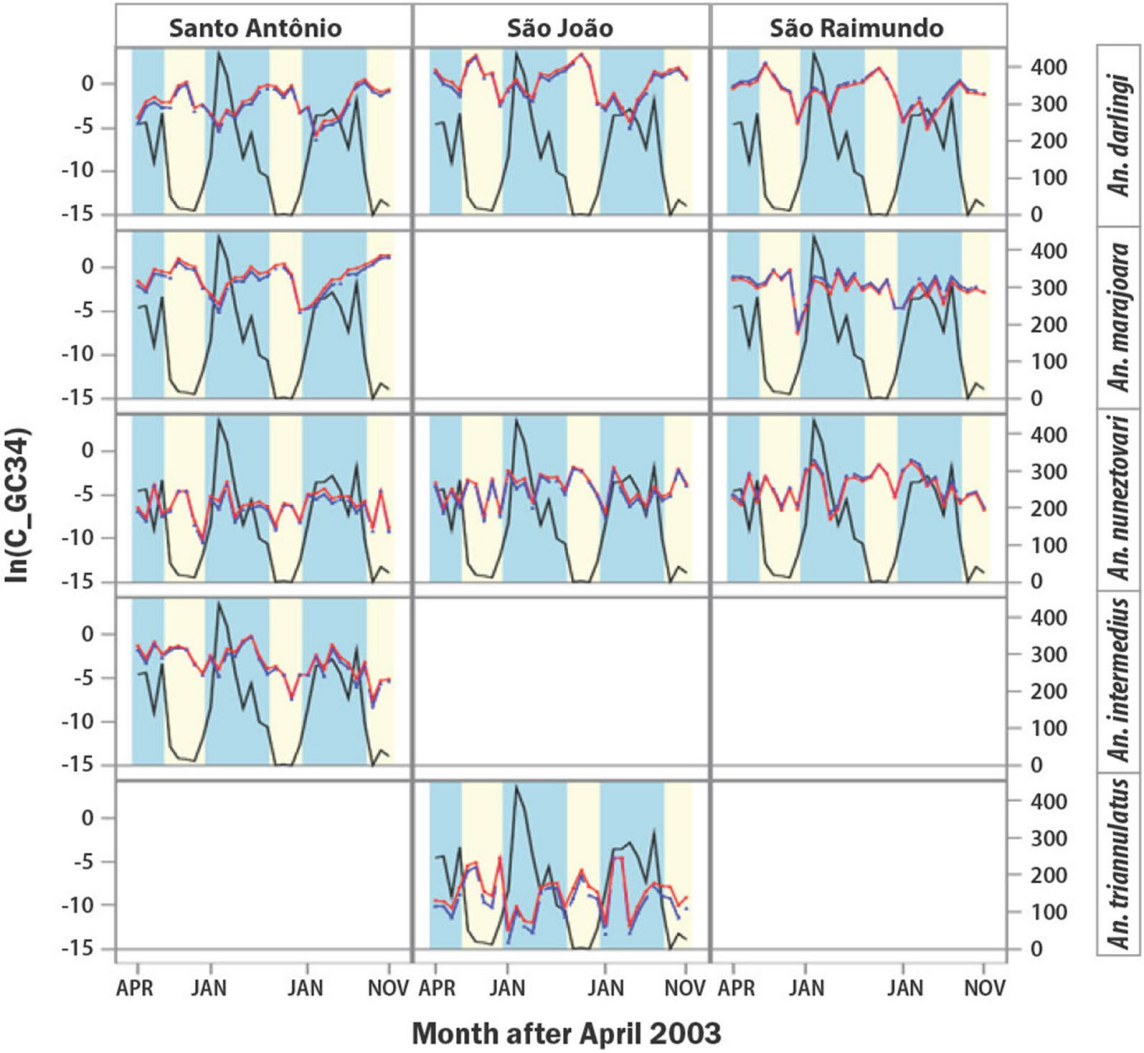
Monthly rainfall (mm) and vectorial capacity (C) with gonotrophic cycle (GC) of 3.4 days by vector species in the three villages. C on y-axis is in log base e scale. Blue lines = *P. falciparum*, Red line = *P. vivax*, Black line = rainfall (mm); Blue bars = rainy season, Yellow bars = dry season

**Fig. 4:**
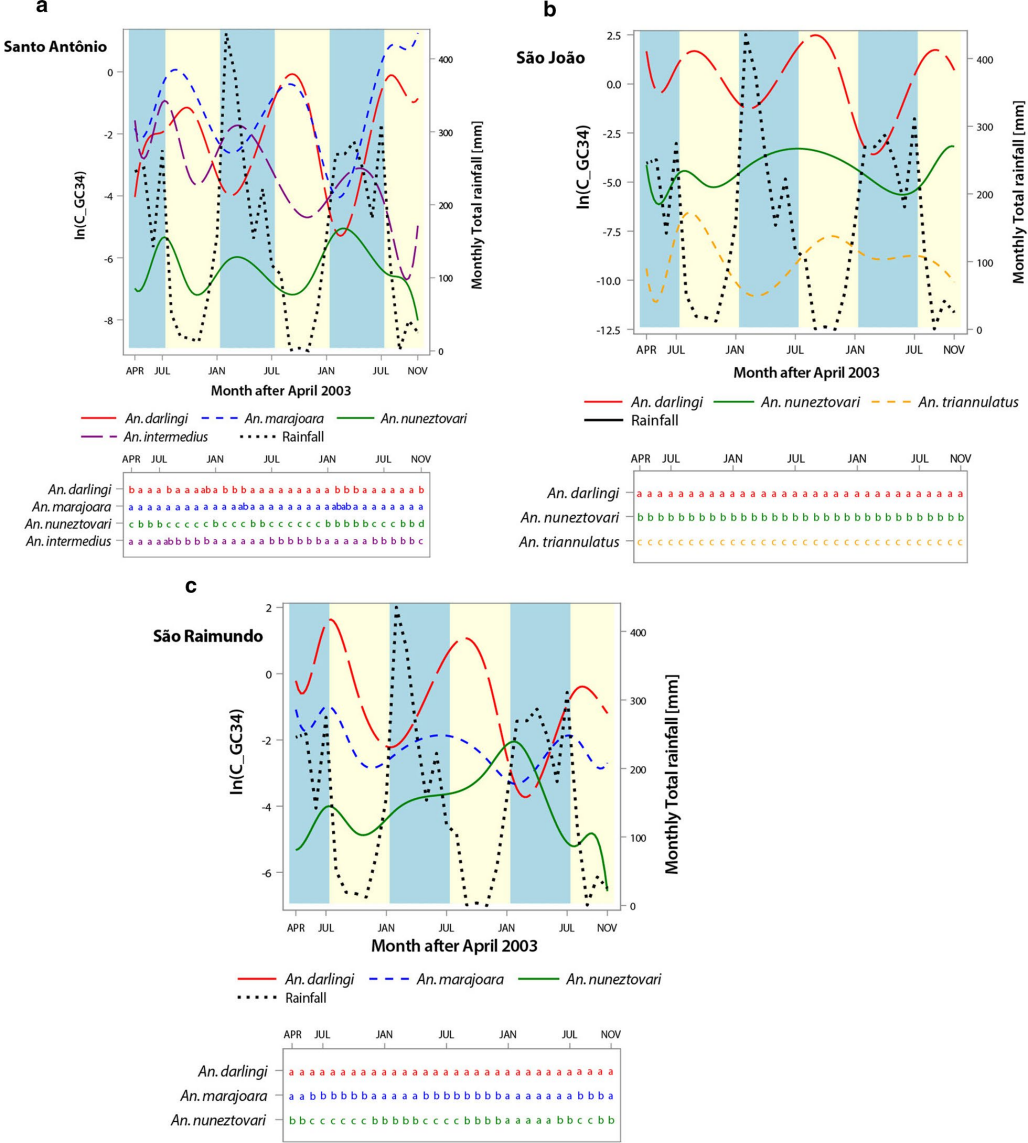
Average vectorial capacities (C), across the two parasites, of *Anopheles* species using a gonotrophic cycle (GC) of 3.4 days displayed to emphasize seasonal effects. C on y-axis is in log base e scale. Blue bars = rainy season; Yellow bars = dry season. Successive panels portray results in different villages, as labelled. Different letters in a column beneath each panel denote significant differences (p < 0.01) in interspecies comparisons of C

A summary of the means (x̅) and the 95% confidence intervals (CI) of the vectorial capacities for *An. darlingi*, *An. marajoara* and *An. nuneztovari* over the 32-month study is presented in Table 4. Means were determined using the different monthly duration of the sporogonic cycle in days (n) for each parasite species. Figure 3 shows the monthly C values with GC of 3.4 days by parasite and vector species in each village, and Fig. 4 demonstrates the monthly averages across the two parasites for C by village. The averaging in Fig. 4 was made possible because of the non-significant parasite × vector species interaction in the GLMM analysis (Table 5).

**Table 4:** Means (x̅) and the lower and upper bounds of the 95% Confidence Intervals (CI) of the vectorial capacities of *An. darlingi*, *An. marajoara* and *An. nuneztovari* calculated using the different monthly durations of the sporogonic cycle in days (n) for *P. falciparum* and *P. vivax* in each village

	Santo Antônio		São João		São Raimundo	
	x̅	95%CI	x̅	95%CI	x̅	95%CI
*P. falciparum*						
*An. darlingi*	0.255	0.140, 0.369	3.679	1.480, 5.878	1.106	0.397, 1.813
*An. marajoara*	0.591	0.307, 0.875	–	–	0.169	0.103, 0.236
*An. nuneztovari*	0.003	0.001, 0.004	0.027	0.014, 0.041	0.049	0.024, 0.073
*P. vivax*						
*An. darlingi*	0.361	0.201, 0.519	4.711	2.057, 7.364	1.373	0.558, 2.188
*An. marajoara*	0.863	0.482, 1.243	–	–	0.239	0.157, 0.321
*An. nuneztovari*	0.004	0.001, 0.006	0.036	0.346, 0.377	0.066	0.031, 0.101

**Table 5:** Output from GLMM analyses showing effects of vector and parasite species, their interactions, and effects of spline regressions interacting with vector species on vectorial capacity (C) in each village

Village/Effect	df Num	df Den	F	P > F
Santo Antônio				
Vector species	3	111.1	3.38	0.02
Parasite species	1	23.6	8.89	0.01
VecSp x ParSp	3	17.8	0.03	0.99
SplineRegr x VecSp	36	45.5	14.10	0.00
São João				
Vector Species	2	96.9	2.08	0.13
Parasite Species	1	28.8	7.90	0.01
VecSp x ParSp	2	29.3	0.33	0.72
SplineRegr x VecSp	27	56.3	7.34	0.00
São Raimundo				
Vector Species	2	100.8	1.41	0.25
Parasite Species	1	30.7	4.19	0.05
VecSp x ParSp	2	23.2	0.06	0.99
SplineRegr x VecSp	27	34.2	5.38	0.00

Because gonotrophic cycle durations varied and averaged between 3.4 and 3.5 days, 3.4 days was used for the calculation of the human biting frequency *a* in Garrett-Jones C model (*ma·a·p*^*n*^*/-ln(p*)), and for the survival rate *p* for all five species (*p* = ^*GC*^***√**** parity*). The C for *An. marajoara* was not calculated in São João where no engorged mosquitoes of this species were collected. *Anopheles darlingi* had the highest overall mean C for both *P. falciparum* and *P. vivax* over the 32 months of capture in São Raimundo and São João, and *An. marajoara* had the highest mean C in Santo Antônio. The monthly C values of *An. darlingi* and *An. marajoara* began to increase in the middle of the rainy season and were highest during the dry season from August to December, while *An. nuneztovari* had its highest monthly C values in the rainy season from January to July, and generally decreased in the dry season but not always (Figs. 3, 4).

The vectorial capacities of *An. triannulatus* and *An. intermedius* could be calculated only for these species in São João and Santo Antônio, respectively, because no engorged mosquitoes of these species were collected in the other villages (Figs. 3, 4). *Anopheles triannulatus* had a mean C of 0.003 ± 0.001 with a range of 0.0–0.0036 for *P. falciparum* and a mean C of 0.006 ± 0.001 and a range of 0.0–0.0062 for *P. vivax*. *Anopheles intermedius* had a mean C of 0.01 ± 0.15 with a range of 0.0–0.73 for *P. falciparum* and a mean C of 0.1257 ± 0.1832 and a range of 0.0–0.8616 for *P. vivax*. The vectorial capacity of *An. triannulatus* was greater during the dry season than in the wet season, and greater for *An. intermedius* in the wet season than in the dry season (Figs. 3, 4). Seasonal trends in vectorial capacities of species were not altered when a gonotrophic cycle length of 3.0 days was substituted for 3.4 days (Additional file 4: Figs. S1–S3).

The GLMM analysis applied to the C results from each village, with vector and parasite species and their interactions as dependent variables, detected significant effects of malaria parasite species in all villages, but a significant effect of vector species on C was detected only in Santo Antônio (Table 5). Interactions between the spline regressions and C by species were significant in all three villages (Table 5), presumably attributable to the spline regressions being unique to each species fitted to the time series. Significant interactions between vector and parasite species were not detected, suggesting that fluctuations in these two variables were not linked to influence C.

Following the GLMM analyses of C by village, post-hoc pairwise comparisons of LSMs were applied to detect significant differences in C among the vector species. In Santo Antônio, *An. darlingi* and *An. marajoara* were co-dominant, frequently during the time series, showing no significant differences in C values, while *An. nuneztovari* was least important in vectorial capacity and *An. intermedius* showing intermediate C values (Fig. 4). By contrast, in São João *An. darlingi* consistently had the highest C among the three vector species evaluated in this village over the course of 32 months (Fig. 4), compared to *An. nuneztovari* and *An. triannulatus*. The comparisons of C among vector species in São Raimundo were more similar to Santo Antônio, in the sense that the position of highest C fluctuated between *An. darlingi* and *An. marajoara* across the time series, with *An. nuneztovari* showing comparatively high, and non-significant different C values only late in the time series (Fig. 4).

Results of the analysis using Spearman’s correlation coefficient (*ρ*) indicated that there was a strong to very strong relationship between C and HLC for *An. darlingi* for falciparum and vivax malaria in all three villages, for *An. marajoara* in Santo Antônio, and for *An. triannulatus* in São João (Table 6). *Anopheles marajoara* had a very weak and nonsignificant relationship between C and HLC for *P. falciparum* (*ρ* = 0.156, p = 0.41), and a weak relationship between C and HLC for *P. vivax* (*ρ* = 0.369, p = 0.04) in São Raimundo. The relationship between C and HLC for the two malaria parasites was weak to moderate for *An. intermedius* in Santo Antônio (Table 6).

**Table 6:** Spearman correlation coefficient (*ρ*) assessing the relationship between monthly vectorial capacity (C) and human biting rate (HLC) calculated from the C values for *P. falciparum* and *P. vivax* ^a^

Species	Santo Antônio		São João		São Raimundo	
	falciparum	vivax	falciparum	vivax	falciparum	vivax
*An. darlingi*	0.737	0.797	0.762	0.778	0.779	0.804
*An. marajoara*	0.867	0.886	–	–	0.156	0.369
*An. nuneztovari*	0.797	0.567	0.454	0.537	0.587	0.635
*An. triannulatus*	–	–	0.731	0.767	–	–
*An. intermedius*	0.367	0.452	–	–	–	–

^a^ Correlation coefficient (ρ_30_, p < 0.05), except for *An. marajoara* with *P. falciparum* in São Raimundo (ρ_30,_ p = 0.40) and *An. intermedius* with *P. falciparum* in Santo Antônio (ρ_29,_ p = 0.051)

## Discussion

### General pattern and variability of survival rates

The recapture rates using human landing catches during the MRR studies were low compared to most previous studies, including some of the same malaria vector species [42, 48–50]. For example, Charlwood and Alecrim [33] conducted a mark-recapture study with *An. darlingi* in western Amazonia, Rondonia State, Brazil and had recapture rates between 12 and 19% with only 319 released *An. darlingi*. The recapture rate for other anopheline species in their study was 2.3%. The highest recapture rate in the present study was 2.8% for *An. darlingi*.

The survival rates of anophelines measured by the Fisher-Ford trellis method were mostly lower than those from the Davidson method during the same time period. This result was similar to studies on *An. gambiae *sensu lato using the Fisher-Ford trellis method [49, 50], and the previous authors concluded that the Fisher-Ford trellis method most likely overestimated mortality compared to other methods. This could have occurred in the present study, wherein the low recapture rates may have been affected by the presence of alternative hosts, such as pigs and water buffalo, which were the dominant blood sources for anophelines in São João [28], and the heterogeneous environment around São João. On the other hand, the survivorship estimates from the Davidson method and the time series analysis were similar. In Papua New Guinea, *Anopheles farauti* had higher survival rates when calculated using the Davidson method than the survival rates estimated from the Birley and Boorman [31] time series analysis [41]. The authors suggested that the mean parous rate method over-estimated the survival rate because of the assumption of constant recruitment, while the time series analysis does not assume constant recruitment and therefore may be a better indicator of survival rate. This is in agreement with Holmes and Birley [43] who concluded that unlike parous rate as an estimator of survival rate, time series analysis does not assume a consistent age structure.

Results of the present study were variable, with no direct indication that the Davidson method or the time series analysis was a better estimate of survival rate. However, the variability in the HLC and parity rates over the 32 months of the study showed that the assumption of constant recruitment of mosquitoes was not met. Therefore, the time series analysis of 32 days would be the most reliable method of estimating survivorship and calculating vectorial capacity. In addition, the time series analysis directly estimates the gonotrophic cycle duration of the vector species while the Davidson method does not.

*Anopheles darlingi* and *An. nuneztovari* showed consistently higher or equal survival rates in the dry season compared to the wet season when estimated by the Davidson method during the MRR study, but not by the Fisher-Ford MRR. Nevertheless, the mean survival rates calculated using the 3-day monthly collections for all five species were higher in the dry season than in the wet season, except for *An. intermedius* in Santo Antônio, (Additional file 3: Table 1). These results were similar to others from western Venezuela where *An. nuneztovari* and *An. albitarsis* (= *An. marajoara*) had higher daily survival rates in the dry season compared to the wet season, and *An. triannulatus* had a higher daily survival rate in the dry season compared to the wet season in 1989 [40]. In the present study, significant differences were found in survival among species and villages, and for season x village interactions (Table 2).

Higher survival rates and higher temperatures would favour a greater transmission of malaria during the dry season, which was the case for *P. vivax* at the three villages. There were 136 *P. vivax* cases reported in the three villages during the research study (April 2003–November 2005) of which 80% were recorded in the dry season [8]. Also, the number of *P. vivax* cases reported from 1998 to 2005 showed similar results with 342 cases reported, of which 82% occurred during the dry season. The dry season was when the Cs of the two most important vectors in the region, *An. darlingi* and *An. marajoara* were higher. The C of *An. triannulatus* in São João was also higher during the dry season (Fig. 4). However, 51 of the 68 cases of *P*. *falciparum* occurred in the rainy season, of which 19 cases occurred in Santo Antônio in April 2003 and 13 cases in São Raimundo in June 2003. Also, from 1998 to 2005, 52% of the 136 *P. falciparum* cases occurred in the rainy season. The high C values of *An*. *nuneztovari* and *An*. *intermedius* in the rainy season (Figs. 3, 4), and the increase in C of the other three vectors as the rainy season progressed provided an abundance of vectors that could have contributed to the transmission of *P. falciparum* in the wet season.

During a previous study in the same villages, we found only slight differences in interpopulation infection rates between villages [8], and that the overall sporozoite percentage was higher for *P. falciparum* in the wet season (0.71) than in the dry season (0.35), and slightly higher for *P. vivax* in the wet season (1.49) than in the dry season (1.29) (unpub. data). These differences might help explain the higher number of cases of *P. falciparum* in the wet season during this study, but not the higher number of *P. vivax* cases reported in the dry season compared to the wet season. Further studies to address the seasonality of falciparum and vivax malaria transmission are warranted.

### Gonotrophic cycle length

Previous field research on the gonotrophic cycle length has shown that anophelines have variable cycles, ranging from 2 to 4 days even within the same species, and the cycle length can depend on the surrounding habitat [42, 48, 51]. The gonotrophic cycle length of *An*. *darlingi* averaged 3.4 ± 0.06 days and was higher than previous estimates of 2.3 to 3 days for this species [32, 33, 39]. *Anopheles marajoara* had a mean cycle length of 3.42 ± 0.21 days and *An. nuneztovari* had a mean cycle length of 3.5 ± 0.02 days, which were lower than the 4 days estimated for these species in Venezuela [40]. This is the first report of the gonotrophic cycle length of *An. intermedius* (3.49 ± 0.07 days). The similarity of gonotrophic cycle lengths would suggest that the habitat around São João was heterogeneous and provided the necessary environment and hosts for all four vectors during the 32-day capture study.

In the MRR experiments, 11.2%–13.8% of the mosquitoes recaptured had previously taken a partial blood meal, indicating that they took blood the night before or they took more than one blood meal during the same night. This is quite possible since both the pigs and water buffalo around the village of São João exhibited host defensive behaviour, mainly by shaking or moving, to interrupt blood-feeding. Also, twenty-two percent of the engorged mosquitoes captured resting under houses or in the vegetation in São João had mixed blood in their abdomens [28]. An unresolved question is whether there was interrupted feeding, or gonotrophic discordance. Gonotrophic concordance, in which one blood meal results in the maturation of one batch of eggs has been shown to occur in some laboratory studies of neotropical malaria vector species [22, 52], however de Oliveira et al. [53] found that some *An. darlingi* fed more than once in a gonotrophic cycle (gonotrophic discordance). A second pregravid blood meal has been recorded in field studies of several other New World anophelines including *An. albimanus* [54] and *An. vestitipennis* [55] in Mexico. It is impossible to say whether or not the vectors in this study exhibited interrupted feeding or took more than one blood meal during the first gonotrophic cycle as pregravids. Either way, the importance of a proportion of the population taking a second blood meal soon after the first one may increase transmission potential [56] and the expectation of life [17]. However, for the purposes of the present study, changes in the gonotrophic cycle length by 0.4 days would not alter the principal conclusions about vectorial capacity heterogeneities.

### Vectorial capacity (C)

Differences in the components of C impacted the results between villages and parasite species, and among vector species. The spatial and temporal variability of three components of C (*ma*, *a,* and *p*) is summarized in Table 7. The C-values are most prone to changes in HBI (*a*) because it is a squared term, which is also included in HLC (*ma*), and survivorship (*p*) because it is raised to the power of *n* [18, 57]. A gonotrophic cycle of 3 days was also analysed and the results were similar to 3.4 days (Additional file 4: Figs. S1–S3). Mean monthly temperatures influenced the sporogonic period, and therefore, along with the HBI and survival rates, influenced the monthly C. For example, *An. darlingi* had the highest C in São João, with a maximum of 31.5 for vivax and 26.68 for falciparum in October 2004 when the monthly mean temperature was the highest at 29.1 °C, and the duration of the sporogonic cycles (*n*) was the lowest (8.5 for falciparum, 7.2 for vivax). Higher mean temperatures normally occurred in the dry season than in the wet season (x̅** = **28.5 °C in the dry season; x̅ = 26.5 °C in the wet season), which decreased the length of the sporogonic period, thereby affecting C. Presumably, the different responses of the two malaria parasite species to temperature, differentially affecting sporogonic cycle lengths, account for the significant effects of parasite species on C within the study villages.

**Table 7:** Spatial and temporal comparison among vector species of variable parameters of C

Vector species	Daily survivorship^1^ (*p*)	HBI^2^ (*a*)	Abundance^2^ (HLC) (*ma*)
*An. darlingi*	SR (0.84) > SJ(0.81) > SA(0.79) Dry(0.83) > Wet(0.79)	SJ(0.35) > SR(0.25) > SA(0.05)	SA(837.6) > SJ(130.8) > SR(54.4)
*An. marajoara*	SR(0.79) > SJ(0.77) > SA(0.76) Dry (0.80) > Wet(0.75)	SR(0.22) > SA(0.14) > SJ(0.00)	SA(316.2) > SJ(58.4) > SR(41.9)
*An. nuneztovari*	SR(0.82) > SJ(0.79) ~ SA(0.79) Dry (0.82) > Wet(0.78)	SR(0.08) > SJ(0.04) > SA(0.02)	SJ(123.7) > SA(43.1) > SR(30.3)
*An. triannulatus*	SA(0.74) > SJ(0.69) ~ SR(0.69) Dry(0.73) > Wet(0.67)	SJ(0.03) > SA(0.00),SR(0.00)	SA(34.8) > SJ(8.8) > SR(4.1)
*An. intermedius*	SR(0.82) > SJ(0.78) ~ SA(0.78) Dry(0.82) > Wet(0.77)	SA(0.10) > SJ(0.00)	SA(42.9) > SJ(30.2) > SR(8.6)

Villages represented by two-letter abbreviations of their names

^1^Values derived from (Additional file 3: Table S1)

^2^Values from [28]

The HLC differed with a mean of 40 for *An. darlingi* collected in São Raimundo, 291 in São João and 1297 in Santo Antônio. One might think that C would be highest in Santo Antônio and lowest in São Raimundo, but it was not. This was in part attributed to the differences in the HBI for this species, which was highest in São João (0.35) followed by São Raimundo (0.25) and Santo Antônio (0.05) (Table 7) and [28]. Therefore, even though the largest number of *An. darlingi* collected landing on humans was in Santo Antônio throughout the study (Table 7), the probability of biting humans was the lowest in that village. The unintentional zooprophylaxis from water buffalo being moved by villagers into corrals next to the village each evening in Santo Antônio provided an alternative host for human malaria vectors. Seventy-seven percent of the engorged *An. darlingi* collected in Santo Antônio fed on water buffalo [28]. This result supports the examination of the individual parameters of vectorial capacity where in theory the diversion of blood meals from humans to non-human hosts would reduce C by a significant amount [58]. In addition, models developed by Saul [59] demonstrated that for areas where malaria is endemic and the human blood index is low, the presence of alternative animal hosts would reduce the vectorial capacity and the human inoculation rate.

Also, the daily survival rate of *An. darlingi* in October 2004 in Santo Antônio was the lowest (0.73) compared to São João (0.88), and São Raimundo (0.93) (Table 7). A contrary view that decreased transmission may be offset by vector survival by feeding on alternative hosts [59], or when vector density was high [16], was not the case in this study. Differences in survival rates and HBI impacted C even in villages within a few kilometers of each other.

The seasonality of the vectorial capacities (Figs. 2, 3) was similar to the seasonal abundance of vector species [14], with *An. darlingi*, *An. marajoara* and *An. triannulatus* showing the highest C values and relative abundances at the end of the wet season into the dry season. *Anopheles nuneztovari* had higher C and seasonal abundance during the wet season in Santo Antônio and São Raimundo, but in São João there was also an increase in C in the dry season for this species, but not in abundance. This may have been due to the sudden increase in survival rate of this species from 0.72 in August to an average of 0.88 from October to December 2004, and a similar increase in 2005 from 0.76 in September to an average of 0.90 in October–November. The C and seasonal abundance of *An. intermedius* were highest in the wet season (Figs. 2, 3, and [14]). The fidelity of the seasonal peaks and valleys of C values across three cycles of wet-dry transitions in all three villages (Fig. 4) supports the premise that C is strongly regulated by, or concordant with, local weather and ecological surroundings. The significant differences between season, among vector species, among villages, and the significant season X village interaction in the 3-way ANOVA analysis of survivorship (Table 2), suggests that temporal, spatial, and species-specific variations in the strength of these three factors impact variations in C. The higher dry-season values of C for the major vector species *An. darlingi* and *An. marajoara* suggest that their superior survivorship in the dry season may contribute more to vectorial capacity than it does in the less abundant vector species, such as *An. intermedius* and *An. nuneztovari*.

The parasite species were found to be significant sources of variation for C in all villages (Table 5) and this is the first report from field estimates showing that vectorial capacities differ among *Anopheles* spp. for transmission of *P. vivax* and *P. falciparum.*

The vectorial capacities reported for the anopheline species in the present study were the first estimates in the Amazon region of Brazil using the equation of Garrett-Jones. An alternative to the vectorial capacity formula was used to estimate the C for the transmission of *P. vivax* by *Ny. darlingi* (= *An. darlingi*) elsewhere in Amazonian Brazil [27]. The results of that study relied heavily on malaria epidemiology to estimate vector competence, basic reproduction number, and alternatives to the entomological parameters, such as vector survivorship, of the Garrett-Jones equation. Moreover, the vectorial capacities estimated by Sallum et al. [27] presume that this species was the unique vector of *P. vivax* at the six sites studied, an assumption that could be validated only by more intensive entomological sampling. The resulting C of Sallum et al. [27] should not be compared to the C values in the present study, and the prominent impacts of survivorship in C calculations in this study calls into question any estimates of vectorial capacities that do not include vector survivorship.

In Africa, in the village of Sugungum, located in the savanna zone of northern Nigeria where malaria transmission is intense, the estimated C-values with *An. gambiae* and *An. funestus* for *P. falciparum* malaria showed an increase during the rainy season, reaching above 35 [17, 60]. Also, in Kankiya, Nigeria a peak C of 16.2 for *An. gambiae* was estimated during the rainy season [61]. Comparable C values were found for *An. darlingi* and falciparum malaria in São João, but in the dry season; reaching 22.24 in September 2003 and 26.68 in October 2004 (Fig. 3). The C values for *An. darlingi* and vivax malaria were highest during the same months, 27.38 and 31.51, respectively. If the results for the five vectors were combined, the C would be even higher. Wet season C values were much lower in the present study area than in Nigeria. The vector dynamics, and the ecosystem in Nigeria were very different from those in this study [14, 60, 61]. For example, the ecosystem in Brazil was a mixed flooded forest, marsh habitat with many small streams, while the sites in Nigeria were in a sub-Saharan savanna. These factors probably contributed to the differences in wet and dry season C values between the two research areas.

In other countries including some in Africa, the C values for the principal vector species were not as high as for *An. darlingi* in São João and were generally similar to those from São Raimundo and Santo Antônio [40, 61–68]. A comparative analysis of the C values and parameters measured in these and other studies is recommended. Vectorial capacity and its components play a vital role in evaluating vector control measures and malaria elimination.

The search for fewer entomological predictors of malaria incidence is warranted, however the present study demonstrated that all the components of C were important. Previously, HLC values were shown to be correlated with malaria incidence [14] and in the present study HLC were correlated with C (Table 6), but *p*, *n*, and HBI also impacted C. Comparative studies of C in similar and different geographical regions, landscapes, and epidemiological situations are warranted to compare the weights of the components of C. Operational studies that include different (or changing) HBI, and pre-post implementation of control measures to evaluate the importance of C and its components in multi-vector settings are warranted.

The major significance of this study was that it took place in three villages over 32 consecutive months. Also, the comparison of the three methods of measuring survival indicated that a time series analysis would most likely be the best method to use when comparing different species survival rates and gonotrophic cycles. However, in practical terms for monitoring and evaluating vector control interventions, Davidson’s method may be the method of choice, after one has a reliable method of determining the length of the gonotrophic cycle. The major limitation in this study was the inability to collect many engorged mosquitoes. For example, *An*. *marajoara* was collected in abundance in São João [14] and was considered the most important vector in the region along with *An. darlingi* [8], but because no engorged *An*. *marajoara* were collected in São João, its HBI or C could not be calculated. In Garrett-Jones’ vectorial capacity model (*ma*^*2*^*p*^*n*^*/-ln(p*)), HLC (*ma*) is multiplied by HBI (*a*), therefore since HBI was not calculated, the vectorial capacity of *An*. *marajoara* could not be compared to the C values of other species collected in São João. The number of engorged mosquitoes collected might be improved by using barrier screens [69], which were used for collecting engorged *An. darlingi* in Peru [70].

The epidemiology of malaria in the study area has not changed significantly from 2005 until today. Malaria cases have fluctuated from year to year and have increased recently in the northern part of Amapà State, but not in the study area [71]. *Plasmodium vivax* is the most prevalent human malaria in the area, and landscape changes have been minimal along the Matapì River and in the riverine communities studied.

## Conclusions

The comprehensive analyses of entomological and parasitological variables revealed hidden complexities of malaria epidemiology at local scales in rural riverine villages of the Amazon Region.

This study demonstrated that local conditions need to be taken into consideration when determining what would be the most appropriate intervention where multiple vectors are involved and malaria is an important health problem. Change in host availability and environmental conditions that affect vector presence, abundance, and survival are key factors to consider in the timing of interventions. The presence of more than one vector species with different host preferences, host-seeking behaviours, larval habitats, resting behaviours (indoors vs. outdoors), and repellency to insecticides (i.e., indoor wall spraying and impregnated bed-nets) will impact the type and timing of interventions. The different seasonal weights of the components of C also can be factored into vector control strategies. For example, the higher dry season survivorship of most vector species analysed in this study would favour an intervention to reduce adult female survivorship, such as entomopathogenic fungi [72], but the greater weight of vector biting densities in the wet season might argue for source reduction strategies during the rainier periods.

A comprehensive analysis of the local and regional conditions that affect malaria transmission and its variability, including a vector(s)’s capacity to transmit malaria, would instruct and help develop flexible control programs based on the changing, dynamic pattern of malaria transmission in the Amazon Region.

## Supplementary Information

**Additional file 1:** Tables of mark-release-recapture results for An. darlingi, An. marajoara and An. nuneztovari.

**Additional file 2:** Comparison of monthly parity and survival rates of Anopheles species. Parity and survival rates were not estimated for months with less than 10 mosquitoes. A gonotrophic cycle of 3.4 days was used to calculate survival rates.

**Additional file 3:** Comparison of survival rates by village, species and season.

**Additional file 4:** Figures comparing the vectorial capacities (C) of Anopheles species using a gonotrophic cycle (GC) of 3 days.

## Data Availability

The datasets used and/or analysed during the current study are available from the corresponding author on reasonable request.

## Abbreviations

C: Vectorial capacity
HLC: Human landing catch
HBI: Human blood index
PR: Parous rate
GC: Duration of the gonotrophic cycle
LSD: Least significant difference
LSMs: Least square means
P: Survival rate for the mean gonotrophic period
u: Estimated gonotrophic cycle duration in days
p: Daily survival rate
n: Duration of the sporogonic cycle in days
CCI: Cross correlation index
MAI: Month after the initiation (of collections)
MRR: Mark-release-recapture
ANOVA: Analysis of variance
GLMM: Generalized linear mixed model
