# Supplementary material for: Vectorial capacities for malaria in eastern Amazonian Brazil depend on village, vector species, season, and parasite species
Source: Malar J. 2022 Aug 16;21:237. doi: 10.1186/s12936-022-04255-x (PMC9382821; doi:10.1186/s12936-022-04255-x)
Supplement: Supplementary file 1 — Additional file 1: Tables of mark-release-recapture results for An. darlingi, An. marajoara and An. nuneztovari. [file 12936_2022_4255_MOESM1_ESM.docx]

**Additional file 1: Tables of mark-release-recapture results for *An. darlingi*, *An. marajoara* and *An. nuneztovari***

| **May-Jun 2004** | **No. released** | **Recaptures** | | | | | | | | | | | **Total**  **collected** | | **% Recaptured** | |
| --- | --- | --- | --- | --- | --- | --- | --- | --- | --- | --- | --- | --- | --- | --- | --- | --- |
| ***An. darlingi*** | |  |  |  |  |  |  |  |  |  |  |  | |  | |  |
| Date |  | 31 May | 1 Jun | 2 Jun | 3 Jun | 4 Jun | 5 Jun | 6 Jun | 7 Jun | 8 Jun | 9 Jun |  | |  | |  |
| 31-May | 187 |  | 1 | 1 | 1 | 0 | 0 | 0 | 0 | 0 | 0 | 3 | | 1.6 | |  |
| 1-Jun | 297 |  |  | 4 | 1 | 2 | 0 | 0 | 0 | 0 | 0 | 7 | | 2.4 | |  |
| 2-Jun | 209 |  |  |  | 0 | 1 | 2 | 0 | 0 | 0 | 0 | 3 | | 1.4 | |  |
| 3-Jun | 154 |  |  |  |  | 1 | 1 | 0 | 0 | 0 | 0 | 2 | | 1.3 | |  |
| 4-Jun | 365 |  |  |  |  |  | 2 | 4 | 0 | 1 | 0 | 7 | | 1.9 | |  |
| Total | 1212 |  | 1 | 5 | 2 | 4 | 5 | 4 | 0 | 1 | 0 | 22 | | 1.8 | |  |
| Captures |  | 199 | 360 | 225 | 177 | 365 | 137 | 245 | 155 | 197 | 341 | 2401 | |  | |  |
| ***An. marajoara*** | |  |  |  |  |  |  |  |  |  |  |  | |  | |  |
| Date |  | 31 May | 1 Jun | 2 Jun | 3 Jun | 4 Jun | 5 Jun | 6 Jun | 7 Jun | 8 Jun | 9 Jun |  | |  | |  |
| 31-May | 716 |  | 6 | 1 | 1 | 2 | 0 | 0 | 0 | 0 | 0 | 10 | | 1.4 | |  |
| 1-Jun | 1066 |  |  | 6 | 0 | 0 | 0 | 0 | 0 | 0 | 0 | 6 | | 0.6 | |  |
| 2-Jun | 487 |  |  |  | 0 | 3 | 0 | 0 | 0 | 0 | 2 | 5 | | 1.0 | |  |
| 3-Jun | 352 |  |  |  |  | 3 | 1 | 1 | 0 | 0 | 1 | 6 | | 1.7 | |  |
| 4-Jun | 1145 |  |  |  |  |  | 9 | 3 | 0 | 2 | 1 | 15 | | 1.3 | |  |
| Total | 3766 |  | 6 | 7 | 1 | 8 | 10 | 4 | 0 | 2 | 4 | 42 | | 1.1 | |  |
| Captures |  | 749 | 1240 | 586 | 415 | 1145 | 685 | 564 | 208 | 1190 | 1282 | 8064 | |  | |  |
| ***An. nuneztovari*** | |  |  |  |  |  |  |  |  |  |  |  | |  | |  |
| Date |  | 31 May | 1 Jun | 2 Jun | 3 Jun | 4 Jun | 5 Jun | 6 Jun | 7 Jun | 8 Jun | 9 Jun |  | |  | |  |
| 31-May | 375 |  | 0 | 0 | 9 | 1 | 0 | 0 | 1 | 0 | 0 | 11 | | 2.9 | |  |
| 1-Jun | 1350 |  |  | 0 | 1 | 2 | 0 | 0 | 0 | 0 | 0 | 3 | | 0.2 | |  |
| 2-Jun | 1189 |  |  |  | 2 | 1 | 0 | 1 | 1 | 2 | 2 | 9 | | 0.8 | |  |
| 3-Jun | 1637 |  |  |  |  | 2 | 0 | 1 | 0 | 1 | 2 | 6 | | 0.4 | |  |
| 4-Jun | 1510 |  |  |  |  |  | 0 | 3 | 1 | 3 | 4 | 11 | | 0.7 | |  |
| Total | 6061 |  | 0 | 0 | 12 | 6 | 0 | 5 | 3 | 6 | 8 | 40 | | 0.7 | |  |
| Captures |  | 395 | 1459 | 1259 | 1746 | 1510 | 558 | 409 | 276 | 1010 | 1088 | 9710 | |  | |  |

**Table S1. Mark-release-recapture results for *An. darlingi*, *An. marajoara*, and *An. nuneztovari*, May-June 2004**

| **October**  **2004** | **No. released** | **Recaptures** | | | | | | | | | | **Total**  **collected** | **%**  **Recaptured** |
| --- | --- | --- | --- | --- | --- | --- | --- | --- | --- | --- | --- | --- | --- |
| ***An. darlingi*** | |  |  |  |  |  |  |  |  |  |  |  |  |
| Date |  | 26 Out | 27 Out | 28 Out | 29 Out | 30 Out | 31 Out | 1 Nov | 2 Nov | 3 Nov | 4 Nov |  |  |
| 26 Oct | 259 |  | 0 | 1 | 2 | 0 | 0 | 0 | 0 | 0 | 0 | 3 | 1.2 |
| 27 Oct | 263 |  |  | 5 | 1 | 1 | 2 | 0 | 0 | 2 | 0 | 11 | 4.2 |
| 28 Oct | 307 |  |  |  | 3 | 3 | 2 | 1 | 1 | 0 | 0 | 10 | 3.3 |
| 29 Oct | 175 |  |  |  |  | 0 | 0 | 1 | 1 | 0 | 0 | 2 | 1.1 |
| 30 Oct | 164 |  |  |  |  |  | 2 | 1 | 0 | 0 | 0 | 3 | 1.8 |
| Total | 1168 |  | 0 | 6 | 6 | 4 | 6 | 3 | 2 | 2 | 0 | 29 | 2.5 |
| Captures |  | 276 | 373 | 332 | 206 | 183 | 140 | 164 | 145 | 138 | 100 | 2057 |  |
| ***An. marajoara*** | |  |  |  |  |  |  |  |  |  |  |  |  |
| Date |  | 26 Out | 27 Out | 28 Out | 29 Out | 30 Out | 31 Out | 1 Nov | 2 Nov | 3 Nov | 4 Nov |  |  |
| 26 Oct | 144 |  | 1 | 0 | 0 | 0 | 0 | 0 | 0 | 0 | 0 | 1 | 0.7 |
| 27 Oct | 233 |  |  | 1 | 1 | 0 | 0 | 0 | 0 | 0 | 0 | 2 | 0.9 |
| 28 Oct | 53 |  |  |  | 2 | 0 | 0 | 0 | 0 | 0 | 0 | 2 | 3.8 |
| 29 Oct | 43 |  |  |  |  | 0 | 1 | 0 | 0 | 0 | 0 | 1 | 2.3 |
| 30 Oct | 18 |  |  |  |  |  | 0 | 0 | 0 | 0 | 0 | 0 | 0.0 |
| Total | 491 |  | 1 | 1 | 3 | 0 | 1 | 0 | 0 | 0 | 0 | 6 | 1.2 |
| Captures |  | 153 | 297 | 77 | 57 | 22 | 9 | 28 | 35 | 18 | 13 | 709 |  |
| ***An. nuneztovari*** | |  |  |  |  |  |  |  |  |  |  |  |  |
| Date |  | 26 Out | 27 Out | 28 Out | 29 Out | 30 Out | 31 Out | 1 Nov | 2 Nov | 3 Nov | 4 Nov |  |  |
| 26 Oct | 422 |  | 1 | 1 | 0 | 0 | 0 | 0 | 0 | 0 | 0 | 2 | 0.5 |
| 27 Oct | 1334 |  |  | 3 | 0 | 0 | 0 | 0 | 0 | 1 | 0 | 4 | 0.3 |
| 28 Oct | 481 |  |  |  | 1 | 0 | 0 | 0 | 1 | 0 | 0 | 2 | 0.4 |
| 29 Oct | 267 |  |  |  |  | 1 | 0 | 0 | 0 | 0 | 0 | 1 | 0.4 |
| 30 Oct | 202 |  |  |  |  |  | 0 | 0 | 0 | 0 | 0 | 0 | 0.0 |
| Total | 2706 |  | 1 | 4 | 1 | 1 | 0 | 0 | 1 | 1 | 0 | 9 | 0.3 |
| Captures |  | 439 | 2233 | 551 | 339 | 230 | 118 | 204 | 249 | 70 | 190 | 4623 |  |

**Table S2. Mark-release-recapture results for *An. darlingi, An. marajoara, and An. nuneztovari*, October 2004**

**Table S3. Mark-release-recapture results for *An. darlingi, An. marajoara, and An. nuneztovari*, June 2005**

| **June**  **2005** | **No. released** | **Recapture** | | | | | | | | | | **Total**  **collected** | **% Recaptured** |
| --- | --- | --- | --- | --- | --- | --- | --- | --- | --- | --- | --- | --- | --- |
| ***An. darlingi*** | |  |  |  |  |  |  |  |  |  |  |  |  |
| Date |  | 21 Jun | 22 Jun | 23 Jun | 24 Jun | 25 Jun | 26 Jun | 27 Jun | 28 Jun | 29 Jun | 30 Jun |  |  |
| 21 Jun | 176 |  | 2 | 3 | 1 | 0 | 0 | 0 | 0 | 0 | 0 | 6 | 3.4 |
| 22 Jun | 171 |  |  | 4 | 2 | 1 | 0 | 0 | 0 | 0 | 0 | 7 | 4.1 |
| 23 Jun | 177 |  |  |  | 2 | 0 | 1 | 1 | 0 | 0 | 0 | 4 | 2.3 |
| 24 Jun | 141 |  |  |  |  | 1 | 0 | 1 | 0 | 0 | 0 | 2 | 1.4 |
| 25 Jun | 128 |  |  |  |  |  | 2 | 0 | 1 | 0 | 0 | 3 | 2.3 |
| Total | 793 |  | 2 | 7 | 5 | 2 | 3 | 2 | 1 | 0 | 0 | 22 | 2.8 |
| Captures |  | 229 | 213 | 204 | 173 | 138 | 161 | 217 | 255 | 361 | 212 | 2163 |  |
| ***An. marajoara*** | |  |  |  |  |  |  |  |  |  |  |  |  |
| Date |  | 21 Jun | 22 Jun | 23 Jun | 24 Jun | 25 Jun | 26 Jun | 27 Jun | 28 Jun | 29 Jun | 30 Jun |  |  |
| 21 Jun | 404 |  | 2 | 2 | 0 | 0 | 0 | 0 | 0 | 0 | 0 | 4 | 1.0 |
| 22 Jun | 218 |  |  | 4 | 2 | 1 | 0 | 0 | 0 | 0 | 0 | 7 | 3.2 |
| 23 Jun | 268 |  |  |  | 8 | 0 | 1 | 0 | 0 | 0 | 0 | 9 | 3.4 |
| 24 Jun | 289 |  |  |  |  | 5 | 0 | 0 | 0 | 0 | 0 | 5 | 1.7 |
| 25 Jun | 253 |  |  |  |  |  | 2 | 0 | 0 | 0 | 0 | 2 | 0.8 |
| Total | 1432 |  | 2 | 6 | 10 | 6 | 3 | 0 | 0 | 0 | 0 | 27 | 1.9 |
| Captures |  | 458 | 282 | 319 | 367 | 289 | 635 | 795 | 446 | 895 | 230 | 4716 |  |
| ***An. nuneztovari*** | |  |  |  |  |  |  |  |  |  |  |  |  |
| Date |  | 21 Jun | 22 Jun | 23 Jun | 24 Jun | 25 Jun | 26 Jun | 27 Jun | 28 Jun | 29 Jun | 30 Jun |  |  |
| 21 Jun | 223 |  | 0 | 4 | 0 | 0 | 0 | 0 | 0 | 0 | 0 | 4 | 1.8 |
| 22 Jun | 194 |  |  | 0 | 2 | 0 | 0 | 0 | 0 | 0 | 0 | 2 | 1.0 |
| 23 Jun | 699 |  |  |  | 0 | 1 | 0 | 0 | 0 | 0 | 1 | 2 | 0.3 |
| 24 Jun | 397 |  |  |  |  | 1 | 2 | 0 | 0 | 0 | 0 | 3 | 0.8 |
| 25 Jun | 304 |  |  |  |  |  | 0 | 0 | 0 | 0 | 0 | 0 | 0.0 |
| Total | 1817 |  | 0 | 4 | 2 | 2 | 2 | 0 | 0 | 0 | 1 | 11 | 0.6 |
| Captures |  | 255 | 249 | 710 | 563 | 329 | 573 | 988 | 701 | 488 | 627 | 5483 |  |

| **September**  **2005** | **No. released** | **Recaptures** | | | | | | | | | | **Total**  **collected** | **% Recaptured** |
| --- | --- | --- | --- | --- | --- | --- | --- | --- | --- | --- | --- | --- | --- |
| ***An. darlingi*** | |  |  |  |  |  |  |  |  |  |  |  |  |
| Date |  | 13 Sep | 14 Sep | 16 Sep | 18 Sep | 20 Sep | 21 Sep | 22 Sep |  |  |  |  |  |
| 13 Sept | 508 |  | 5 | 0 | 0 | 0 | 0 | 0 |  |  |  | 5 | 1.0 |
| 14 Sept | 400 |  |  | 1 | 1 | 0 | 0 | 0 |  |  |  | 2 | 0.5 |
| 16 Sept | 381 |  |  |  | 4 | 0 | 0 | 0 |  |  |  | 4 | 1.1 |
| 18 Sept | 343 |  |  |  |  | 1 | 1 | 0 |  |  |  | 2 | 0.6 |
| 20 Sept | 189 |  |  |  |  |  | 3 | 0 |  |  |  | 3 | 1.6 |
| Total | 1821 |  | 5 | 1 | 5 | 1 | 4 | 0 |  |  |  | 16 | 0.9 |
| Captures |  | 785 | 500 | 550 | 392 | 205 | 580 | 347 |  |  |  | 3359 |  |
| ***An. marajoara*** | |  |  |  |  |  |  |  |  |  |  |  |  |
| Date |  | 13 Sep | 14 Sep | 16 Sep | 18 Sep | 20 Sep | 21 Sep | 22 Sep |  |  |  |  |  |
| 13 Sept | 315 |  | 9 | 1 | 0 | 0 | 0 | 0 |  |  |  | 10 | 3.2 |
| 14 Sept | 238 |  |  | 0 | 0 | 0 | 0 | 0 |  |  |  | 0 | 0.0 |
| 16 Sept | 101 |  |  |  | 1 | 0 | 0 | 0 |  |  |  | 1 | 1.0 |
| 18 Sept | 163 |  |  |  |  | 1 | 0 | 1 |  |  |  | 2 | 1.2 |
| 20 Sept | 108 |  |  |  |  |  | 3 | 2 |  |  |  | 5 | 4.6 |
| Total | 925 |  | 9 | 1 | 1 | 1 | 3 | 3 |  |  |  | 18 | 1.9 |
| Captures |  | 465 | 288 | 179 | 193 | 110 | 320 | 332 |  |  |  | 1887 |  |
| ***An. nuneztovari*** | |  |  |  |  |  |  |  |  |  |  |  |  |
| Date |  | 13 Sep | 14 Sep | 16 Sep | 18 Sep | 20 Sep | 21 Sep | 22 Sep |  |  |  |  |  |
| 13 Sept | 580 |  | 4 | 1 | 0 | 0 | 0 | 0 |  |  |  | 5 | 0.9 |
| 14 Sept | 276 |  |  | 0 | 0 | 0 | 0 | 0 |  |  |  | 0 | 0.0 |
| 16 Sept | 160 |  |  |  | 0 | 0 | 0 | 0 |  |  |  | 0 | 0.0 |
| 18 Sept | 141 |  |  |  |  | 0 | 0 | 0 |  |  |  | 0 | 0.0 |
| 20 Sept | 16 |  |  |  |  |  | 0 | 0 |  |  |  | 0 | 0.0 |
| Total | 1173 |  | 4 | 1 | 0 | 0 | 0 | 0 |  |  |  | 5 | 0.4 |
| Captures |  | 812 | 340 | 243 | 188 | 18 | 224 | 308 |  |  |  | 2133 |  |

**Table S4. Mark-release-recapture results for *An. darlingi, An. marajoara, and An. nuneztovari*, September 2005**
