# Supplementary material for: Vectorial capacities for malaria in eastern Amazonian Brazil depend on village, vector species, season, and parasite species
Source: Malar J. 2022 Aug 16;21:237. doi: 10.1186/s12936-022-04255-x (PMC9382821; doi:10.1186/s12936-022-04255-x)
Supplement: Supplementary file 2 — Additional file 2: Comparison of monthly parity and survival rates of Anopheles species. Parity and survival rates were not estimated for months with less than 10 mosquitoes. A gonotrophic cycle of 3.4 days was used to calculate survival rates. [file 12936_2022_4255_MOESM2_ESM.docx]

**Additional file 2: Comparison of monthly parity and survival rates of *Anopheles* species. Parity and survival rates were not estimated for months with less than 10 mosquitoes. A gonotrophic cycle of 3.4 days was used to calculate survival rates.**

**Table S1. Monthly parity and survival rates of *An. darlingi***

| Rainy Season | | | | | | |  | Dry Season | | | | | | |
| --- | --- | --- | --- | --- | --- | --- | --- | --- | --- | --- | --- | --- | --- | --- |
| Month | São João | | São Raimundo | | Santo Antônio | |  | Month | São João | | São Raimundo | | Santo Antônio | |
|  | Parity | Survival | Parity | Survival | Parity | Survival |  |  | Parity | Survival | Parity | Survival | Parity | Survival |
| April 03 | 55.7 | 0.85 | 55.7 | 0.84 | 24.9 | 0.66 |  | August 03 | 48.6 | 0.81 | 70.3 | 0.90 | 23.2 | 0.65 |
| May | 59.9 | 0.76 | 59.9 | 0.86 | 38.7 | 0.76 |  | September | 58.8 | 0.86 | 49.9 | 0.82 | 39.5 | 0.76 |
| June | 45.8 | 0.72 | 45.8 | 0.79 | 32.5 | 0.72 |  | October | 45.9 | 0.80 | 53.9 | 0.83 | 52.5 | 0.83 |
| July | 43.9 | 0.66 | 43.9 | 0.78 | 30.5 | 0.71 |  | November | 62.4 | 0.87 | 65.6 | 0.88 | 32.4 | 0.72 |
| January 04 | 62.8 | 0.82 | 62.8 | 0.87 | 77.9 | 0.93 |  | December | 51.7 | 0.82 | 40.9 | 0.77 | 78.4 | 0.93 |
| February | 60.2 | 0.83 | 60.2 | 0.86 | 35.0 | 0.73 |  | August 04 | 51.3 | 0.82 | 56.6 | 0.85 | 44.5 | 0.79 |
| March | 65.6 | 0.77 | 65.6 | 0.88 | 53.5 | 0.83 |  | September | 51.1 | 0.82 | 60.3 | 0.86 | 44.6 | 0.79 |
| April | 39.5 | 0.78 | 39.5 | 0.76 | 42.8 | 0.78 |  | October | 64.4 | 0.88 | 79.0 | 0.93 | 34.2 | 0.73 |
| May | 56.5 | 0.86 | 56.5 | 0.85 | 49.9 | 0.82 |  | November | 79.0 | 0.93 | 79.8 | 0.94 | 57.8 | 0.85 |
| June | 48.9 | 0.76 | 48.9 | 0.81 | 37.2 | 0.75 |  | December | 61.1 | 0.87 | 99.0 | 1.00 | 78.4 | 0.93 |
| July | 47.8 | 0.81 | 47.8 | 0.80 | 41.2 | 0.77 |  | August 05 | 52.1 | 0.83 | 52.4 | 0.83 | 44.7 | 0.79 |
| January 05 | 66.7 | 0.79 | 66.7 | 0.89 | 91.7 | 0.97 |  | September | 40.4 | 0.77 | 37.6 | 0.75 | 37.4 | 0.75 |
| February | 50.0 | 0.81 | 50.0 | 0.82 | 37.3 | 0.75 |  | October | 52.7 | 0.83 | 48.3 | 0.81 | 36.7 | 0.74 |
| March | 50.0 | 0.69 | 50.0 | 0.82 | 36.6 | 0.74 |  | November | 58.0 | 0.85 | 62.7 | 0.87 | 57.8 | 0.85 |
| April | 30.2 | 0.66 | 30.2 | 0.70 | 44.8 | 0.79 |  |  |  |  |  |  |  |  |
| May | 40.3 | 0.75 | 40.3 | 0.77 | 40.1 | 0.76 |  |  |  |  |  |  |  |  |
| June | 37.9 | 0.73 | 37.9 | 0.75 | 38.8 | 0.76 |  |  |  |  |  |  |  |  |
| July | 42.2 | 0.85 | 42.2 | 0.78 | 50.3 | 0.82 |  |  |  |  |  |  |  |  |

**Table S2. Monthly parity and survival rates of *An. marajoara***

| Rainy Season | | | | | | |  | Dry Season | | | | | | |
| --- | --- | --- | --- | --- | --- | --- | --- | --- | --- | --- | --- | --- | --- | --- |
| Month | São João | | São Raimundo | | Santo Antônio | |  | Month | São João | | São Raimundo | | Santo Antônio | |
|  | Parity | Survival | Parity | Survival | Parity | Survival |  |  | Parity | Survival | Parity | Survival | Parity | Survival |
| April 03 | 51.8 | 0.82 | 47.9 | 0.81 | 39.5 | 0.76 |  | August 03 | 35.0 | 0.73 | 49.0 | 0.81 | 30.3 | 0.70 |
| May | 38.9 | 0.76 | 61.0 | 0.86 | 44.9 | 0.79 |  | September | 44.1 | 0.79 | 56.1 | 0.84 | 40.9 | 0.77 |
| June | 25.7 | 0.67 | 37.9 | 0.75 | 40.0 | 0.76 |  | October | 31.0 | 0.71 | 57.9 | 0.85 | 30.1 | 0.70 |
| July | 29.6 | 0.70 | 47.0 | 0.80 | 44.7 | 0.79 |  | November | 59.6 | 0.86 | 93.8 | 0.98 | 42.5 | 0.78 |
| January 04 | 26.5 | 0.79 | 33.3 | 0.72 | 43.5 | 0.78 |  | December | - | - | - | - | 49.2 | 0.81 |
| February | 44.3 | 0.75 | 47.0 | 0.80 | 23.8 | 0.66 |  | August 04 | 48.0 | 0.68 | 42.3 | 0.78 | 37.4 | 0.75 |
| March | 37.6 | 0.74 | 35.9 | 0.74 | 44.3 | 0.79 |  | September | 45.0 | 0.81 | 47.0 | 0.80 | 40.1 | 0.76 |
| April | 36.2 | 0.78 | 18.4 | 0.61 | 43.0 | 0.78 |  | October | 68.4 | 0.79 | 46.9 | 0.80 | 37.0 | 0.75 |
| May | 43.5 | 0.77 | 48.0 | 0.81 | 40.8 | 0.77 |  | November | 61.3 | 0.89 | 81.0 | 0.94 | 52.3 | 0.83 |
| June | 41.8 | 0.78 | 30.7 | 0.71 | 39.6 | 0.76 |  | December | - | - | - | - | 54.5 | 0.84 |
| July | 43.7 | 0.80 | 42.4 | 0.78 | 28.5 | 0.69 |  | August 05 | 43.6 | 0.78 | 38.4 | 0.75 | 37.5 | 0.75 |
| January 05 | - | - | - | - | - | - |  | September | 35.0 | 0.73 | 41.1 | 0.77 | 40.5 | 0.77 |
| February | 46.6 | 0.67 | 37.2 | 0.76 | 28.4 | 0.74 |  | October | 44.5 | 0.79 | 50.6 | 0.82 | 45.3 | 0.79 |
| March | 38.2 | 0.77 | 39.3 | 0.66 | 35.5 | 0.75 |  | November | 51.8 | 0.82 | 69.2 | 0.90 | 51.0 | 0.82 |
| April | 25.1 | 0.78 | 24.1 | 0.77 | 38.3 | 0.74 |  |  |  |  |  |  |  |  |
| May | 40.5 | 0.82 | 40.7 | 0.63 | 36.0 | 0.74 |  |  |  |  |  |  |  |  |
| June | 43.0 | 0.82 | 20.7 | 0.75 | 35.6 | 0.73 |  |  |  |  |  |  |  |  |
| July | 50.1 | 0.76 | 37.2 | 0.81 | 34.4 | 0.76 |  |  |  |  |  |  |  |  |

**Table S3. Monthly parity and survival rates of *An. nuneztovari***

| Rainy Season | | | | | | |  | Dry Season | | | | | | |
| --- | --- | --- | --- | --- | --- | --- | --- | --- | --- | --- | --- | --- | --- | --- |
| Month | São João | | São Raimundo | | Santo Antônio | |  | Month | São João | | São Raimundo | | Santo Antônio | |
|  | Parity | Survival | Parity | Survival | Parity | Survival |  |  | Parity | Survival | Parity | Survival | Parity | Survival |
| April 03 | 54.1 | 0.83 | 46.8 | 0.80 | 45.9 | 0.80 |  | August 03 | 53.5 | 0.83 | 73.7 | 0.91 | - | - |
| May | 32.5 | 0.72 | 45.5 | 0.79 | 50.0 | 0.82 |  | September | 42.5 | 0.78 | 47.8 | 0.80 | - |  |
| June | 46.4 | 0.80 | 68.0 | 0.89 | 80.8 | 0.94 |  | October | 23.3 | 0.65 | 40.0 | 0.76 | - |  |
| July | 26.8 | 0.68 | 36.7 | 0.74 | - | - |  | November | 83.0 | 0.95 | 71.4 | 0.91 | 36.4 | 0.74 |
| January 04 | 50.3 | 0.82 | 41.9 | 0.77 | 54.1 | 0.83 |  | December | 34.3 | 0.73 | 30.2 | 0.70 | 28.6 | 0.69 |
| February | 29.4 | 0.70 | 46.7 | 0.80 | 22.7 | 0.65 |  | August 04 | 32.4 | 0.72 | 48.4 | 0.81 | 45.8 | 0.79 |
| March | 42.9 | 0.78 | 56.2 | 0.84 | 56.1 | 0.84 |  | September | 61.8 | 0.87 | 66.7 | 0.89 | - | - |
| April | 28.2 | 0.69 | 26.4 | 0.68 | 29.0 | 0.69 |  | October | 65.9 | 0.88 | 89.2 | 0.97 | 62.1 | 0.87 |
| May | 66.7 | 0.89 | 37.8 | 0.75 | 39.7 | 0.76 |  | November | 63.8 | 0.88 | 85.7 | 0.96 | - | - |
| June | 48.0 | 0.81 | 48.5 | 0.81 | 38.9 | 0.76 |  | December | 69.2 | 0.90 | - | - | - | - |
| July | 46.7 | 0.80 | 49.3 | 0.81 | 49.0 | 0.81 |  | August 05 | 37.3 | 0.75 | 31.5 | 0.71 | 50.0 | 0.82 |
| January 05 | 25.8 | 0.67 | 75.7 | 0.92 | 83.7 | 0.95 |  | September | 40.0 | 0.76 | 61.1 | 0.87 | 32.1 | 0.72 |
| February | 49.6 | 0.81 | 52.7 | 0.83 | 29.0 | 0.69 |  | October | 83.3 | 0.95 | 52.3 | 0.83 | - | - |
| March | 29.3 | 0.70 | 37.8 | 0.75 | 33.0 | 0.72 |  | November | 58.5 | 0.85 | 42.9 | 0.78 | 33.3 | 0.72 |
| April | 34.5 | 0.73 | 51.5 | 0.82 | 36.6 | 0.74 |  |  |  |  |  |  |  |  |
| May | 42.9 | 0.78 | 63.8 | 0.88 | 48.1 | 0.81 |  |  |  |  |  |  |  |  |
| June | 28.6 | 0.69 | 30.7 | 0.71 | 40.6 | 0.77 |  |  |  |  |  |  |  |  |
| July | 46.7 | 0.80 | 40.7 | 0.77 | 33.7 | 0.73 |  |  |  |  |  |  |  |  |

**Table S4. Monthly parity and survival rates of *An. triannulatus***

| Rainy Season | | | | | | |  | Dry Season | | | | | | |
| --- | --- | --- | --- | --- | --- | --- | --- | --- | --- | --- | --- | --- | --- | --- |
| Month | São João | | São Raimundo | | Santo Antônio | |  | Month | São João | | São Raimundo | | Santo Antônio | |
|  | Parity | Survival | Parity | Survival | Parity | Survival |  |  | Parity | Survival | Parity | Survival | Parity | Survival |
| April 03 | 31.6 | 0.71 | 20.0 | 0.71 | - | - |  | August 03 | 43.8 | 0.78 | 57.1 | 0.85 | 38.5 | 0.76 |
| May | 35.0 | 0.73 | 20.0 | 0.73 | 34.9 | 0.82 |  | September | 54.8 | 0.84 | 39.6 | 0.76 | 25.0 | 0.67 |
| June | 13.3 | 0.55 | 16.3 | 0.55 | 31.7 | 0.71 |  | October | 26.1 | 0.67 | 39.1 | 0.76 | 25.0 | 0.67 |
| July | 28.6 | 0.69 | 11.1 | 0.69 | - | - |  | November | 25.0 | 0.67 | 33.3 | 0.72 | 40.5 | 0.77 |
| January 04 | - | - | - | - | - | - |  | December | - | - | - | - | 60.7 | 0.86 |
| February | - | - | - | - | 28.0 | 0.69 |  | August 04 | 15.7 | 0.58 | 67.4 | 0.89 | 36.4 | 0.74 |
| March | - | - | - | - | 46.7 | 0.80 |  | September | 27.9 | 0.69 | 38.2 | 0.75 | 36.1 | 0.74 |
| April | 12.5 | 0.54 | 10.5 | 0.54 | 31.4 | 0.71 |  | October | 49.9 | 0.82 | 51.4 | 0.82 | 26.5 | 0.68 |
| May | 39.6 | 0.76 | 12.5 | 0.76 | 29.3 | 0.70 |  | November | 33.3 | 0.72 | 72.2 | 0.91 | 62.5 | 0.87 |
| June | 40.3 | 0.77 | 34.8 | 0.77 | 29.3 | 0.70 |  | December | 39.3 | 0.76 | - | - | - | - |
| July | 27.4 | 0.68 | 27.8 | 0.68 | 37.0 | 0.75 |  | August 05 | 25.8 | 0.67 | 18.2 | 0.61 | 33.9 | 0.73 |
| January 05 | 16.7 | 0.59 | - | - | - | - |  | September | 21.7 | 0.64 | 30.7 | 0.71 | 13.3 | 0.55 |
| February | - | - | - | - | - | - |  | October | 20.6 | 0.63 | 30.5 | 0.71 | 40.9 | 0.77 |
| March | - | - | - | - | - | - |  | November | 26.3 | 0.68 | 22.2 | 0.64 | 50.0 | 0.82 |
| April | - | - | - | - | - | - |  |  |  |  |  |  |  |  |
| May | 27.3 | 0.68 | 8.7 | 0.68 | 21.9 | 0.64 |  |  |  |  |  |  |  |  |
| June | 27.0 | 0.68 | 13.0 | 0.68 | 20.6 | 0.63 |  |  |  |  |  |  |  |  |
| July | 36.4 | 0.74 | 38.2 | 0.74 | 26.8 | 0.68 |  |  |  |  |  |  |  |  |

**Table S5. Monthly parity and survival rates of *An. intermedius***

| Rainy Season | | | | | | |  | Dry Season | | | | | | |
| --- | --- | --- | --- | --- | --- | --- | --- | --- | --- | --- | --- | --- | --- | --- |
| Month | São João | | São Raimundo | | Santo Antônio | |  | Month | São João | | São Raimundo | | Santo Antônio | |
|  | Parity | Survival | Parity | Survival | Parity | Survival |  |  | Parity | Survival | Parity | Survival | Parity | Survival |
| April 03 | 65.5 | 0.90 | - | - | 45.9 | 0.80 |  | August 03 | 69.8 | 0.91 | 52.9 | 0.83 | 51.1 | 0.82 |
| May | 59.2 | 0.88 | 62.1 | 0.87 | 36.5 | 0.74 |  | September | 45.1 | 0.82 | 62.9 | 0.87 | 66.7 | 0.89 |
| June | 20.2 | 0.67 | 68.7 | 0.90 | 61.7 | 0.87 |  | October | - | - | 81.5 | 0.94 | - | - |
| July | 39.8 | 0.79 | 44.4 | 0.79 | 48.8 | 0.81 |  | November | - | - | - | - | - | - |
| January 04 | 45.8 | 0.84 | 20.3 | 0.63 | 63.1 | 0.87 |  | December | - | - | - | - | 59.1 | 86.0 |
| February | 23.4 | 0.70 | 44.6 | 0.79 | 24.7 | 0.66 |  | August 04 | 54.2 | 0.86 | 70.2 | 0.90 | 27.4 | 0.68 |
| March | 14.9 | 0.62 | 61.6 | 0.87 | 44.7 | 0.79 |  | September | 41.3 | 0.80 | 63.9 | 0.88 | 50.0 | 0.82 |
| April | 23.3 | 0.69 | 34.8 | 0.73 | 41.3 | 0.77 |  | October | - | - | 94.4 | 0.98 | - | - |
| May | 35.0 | 0.77 | 21.0 | 0.63 | 73.1 | 0.91 |  | November | - | - | - | - | - | - |
| June | 66.7 | 0.90 | 50.9 | 0.82 | 73.1 | 0.91 |  | December | - | - | - | - | - | - |
| July | 21.9 | 0.68 | 46.7 | 0.80 | 58.0 | 0.85 |  | August 05 | 53.0 | 0.85 | 67.9 | 0.89 | 35.2 | 0.74 |
| January 05 | - | - | - | - | - | - |  | September | 38.3 | 0.79 | 61.5 | 0.87 | 13.9 | 0.56 |
| February | 33.2 | 0.76 | 35.5 | 0.74 | 70.1 | 0.90 |  | October | 30.0 | 0.74 | 62.1 | 0.87 | 38.5 | 0.76 |
| March | 13.9 | 0.61 | 15.2 | 0.57 | 28.4 | 0.69 |  | November | - | - | 43.9 | 0.78 | 58.0 | 0.85 |
| April | 19.5 | 0.66 | 39.5 | 0.76 | 44.8 | 0.79 |  |  |  |  |  |  |  |  |
| May | 33.5 | 0.76 | 39.7 | 0.76 | 35.9 | 0.74 |  |  |  |  |  |  |  |  |
| June | 29.4 | 0.74 | 52.5 | 0.83 | 31.1 | 0.71 |  |  |  |  |  |  |  |  |
| July | 40.5 | 0.80 | 54.4 | 0.84 | 22.4 | 0.64 |  |  |  |  |  |  |  |  |
