# Supplementary material for: Vectorial capacities for malaria in eastern Amazonian Brazil depend on village, vector species, season, and parasite species
Source: Malar J. 2022 Aug 16;21:237. doi: 10.1186/s12936-022-04255-x (PMC9382821; doi:10.1186/s12936-022-04255-x)
Supplement: Supplementary file 3 — Additional file 3: Comparison of survival rates by village, species and season. [file 12936_2022_4255_MOESM3_ESM.docx]

**Additional file 3 Comparison of survival rates by village, species and season.**

**Table S1. Wet season and dry season mean survival rates by village using the results of 3-day monthly HLC collections**

| **Survival rate** | | | | | |
| --- | --- | --- | --- | --- | --- |
| **Species** | **Dry Season** | | **Wet Season** | |  |
|  | Sample Size | Mean ± SD | Sample Size | Mean ± SD | Difference |
| *An. darlingi* |  |  |  |  |  |
| SR^a^ | 14 | 0.86±0.07 | 18 | 0.81±0.05 | 0.05 |
| SJ | 14 | 0.84±0.04 | 18 | 0.77±0.06 | 0.07 |
| SA | 14 | 0.79±0.08 | 18 | 0.78±0.07 | 0.01 |
| *An. marajoara* |  |  |  |  |  |
| SR | 13 | 0.83±0.07 | 17 | 0.75±0.06 | 0.08 |
| SJ | 14 | 0.79±0.06 | 17 | 0.76±0.06 | 0.03 |
| SA | 14 | 0.77±0.09 | 18 | 0.75±0.08 | 0.02 |
| *An. nuneztovari* |  |  |  |  |  |
| SR | 14 | 0.84±0.08 | 18 | 0.80±0.06 | 0.04 |
| SJ | 14 | 0.82±0.09 | 18 | 0.76±0.06 | 0.06 |
| SA | 11 | 0.79±0.10 | 15 | 0.78±0.08 | 0.01 |
| *An. intermedius* |  |  |  |  |  |
| SR | 10 | 0.87±0.09 | 16 | 0.78±0.08 | 0.09 |
| SJ | 8 | 0.81±0.07 | 17 | 0.75±0.09 | 0.06 |
| SA | 9 | 0.78±0.10 | 17 | 0.79±0.08 | -0.01 |
| *An. triannulatus* |  |  |  |  |  |
| SR | 12 | 0.76±0.09 | 13 | 0.61±0.08 | 0.15 |
| SJ | 12 | 0.70±0.07 | 15 | 0.67±0.07 | 0.03 |
| SA | 13 | 0.74±0.08 | 15 | 0.73±0.08 | 0.01 |

^a^SR = São Raimundo, SJ = São João, SA = Santo Antônio
