# Supplementary material for: Vectorial capacities for malaria in eastern Amazonian Brazil depend on village, vector species, season, and parasite species
Source: Malar J. 2022 Aug 16;21:237. doi: 10.1186/s12936-022-04255-x (PMC9382821; doi:10.1186/s12936-022-04255-x)

**Additional file 4: Figures comparing the vectorial capacities (C) of *Anopheles* species using a gonotrophic cycle (GC) of 3 days**

**Figure S1. Comparison of average vectorial capacities (C), across the two parasites, of four *Anopheles* species using a gonotrophic cycle (GC) of 3 days in Santo Antônio. Blue bars = rainy season; Yellow bars = dry season.**


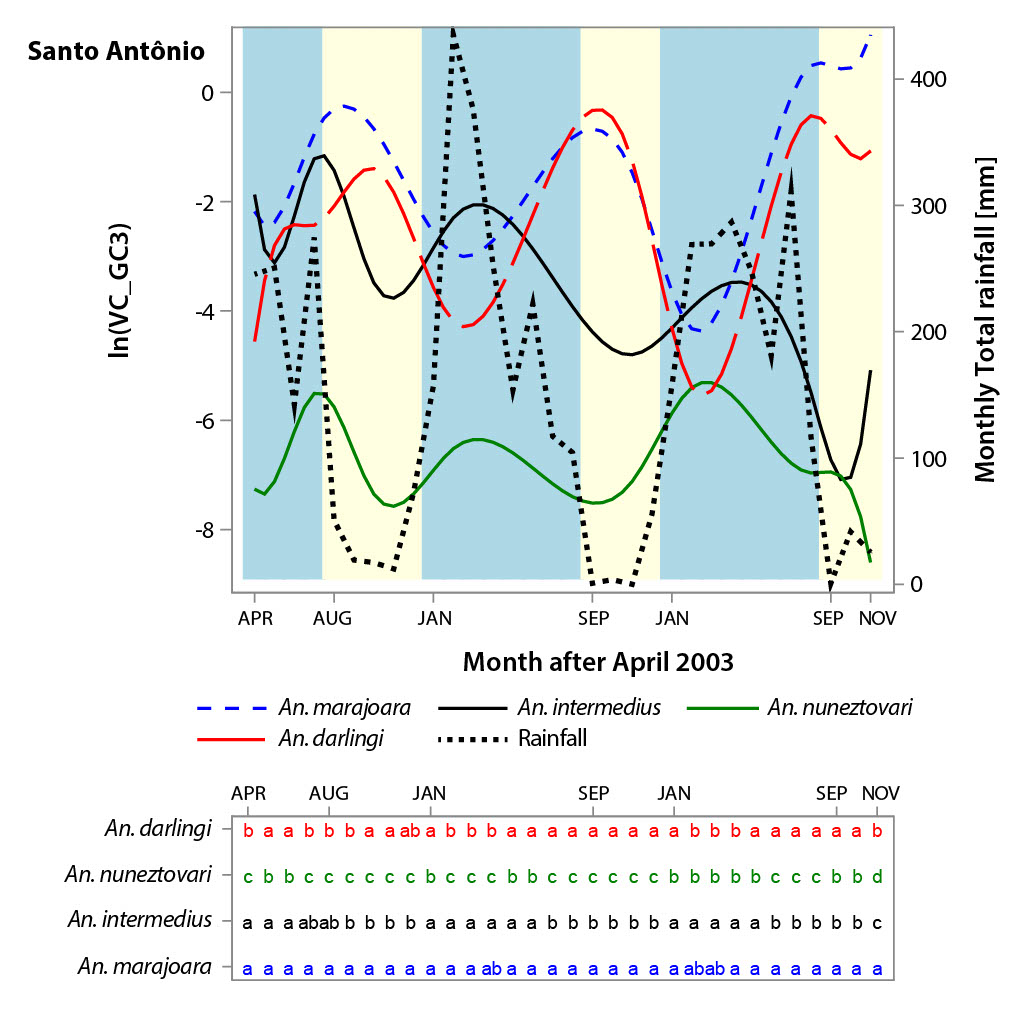


**Figure S2. Comparison of average vectorial capacities (C), across the two parasites, of three *Anopheles* species using a gonotrophic cycle (GC) of 3 days in São João. Blue bars = rainy season; Yellow bars = dry season.**


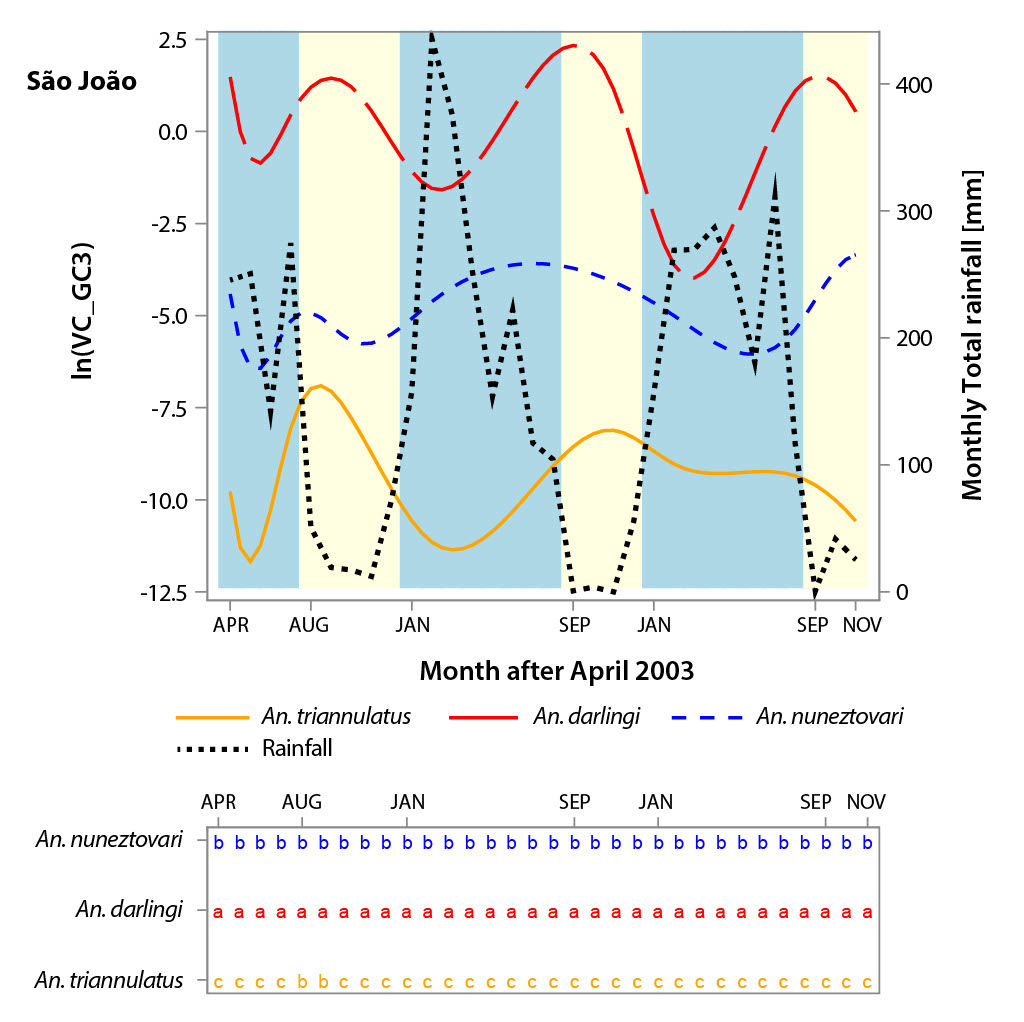


**Figure S3. Comparison of average vectorial capacities (C), across the two parasites, of three *Anopheles* species using a gonotrophic cycle (GC) of 3 days in São Raimundo. Blue bars = rainy season; Yellow bars = dry season.**


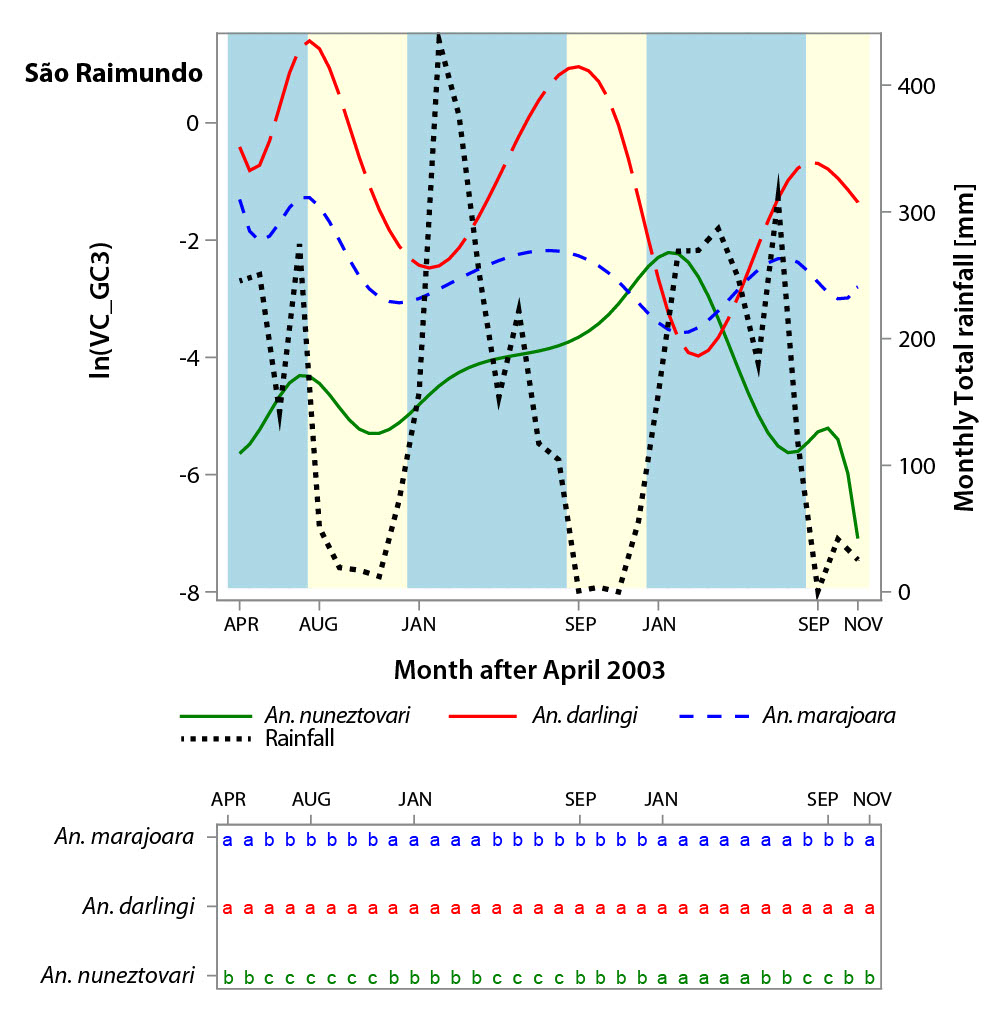

Supplement: Supplementary file 4 — Additional file 4: Figures comparing the vectorial capacities (C) of Anopheles species using a gonotrophic cycle (GC) of 3 days. [file 12936_2022_4255_MOESM4_ESM.docx]
